# Transmission Dynamics of Dengue Virus, Ceará State, Brazil, 2010–2025

**DOI:** 10.3201/eid3211.260609

**Published:** 2026-11

**Authors:** Shirlene T.S. de Lima, Ingra M. Claro, Xinyi Hua, Livia Sacchetto, Ronaldo de Jesus, Leda M. Simões Mello, Júlia Forato, Erika R. Manuli, Cailin McDonald, Maria Eduarda T. de Lima, Clarissa P.M. Ferraz, Larissa M.F. Duarte, Vânia A.F. Viana, Mariana Araújo Costa, Antônio Carlos Firmino, Jayra J.P.A. Abrantes, Ludmila O.C. Sena, Débora Bezerra Silva, Sônia M.S. Macedo, Ester C. Sabino, Rodrigo F. do Carmo, Julio Croda, José Luiz Proenca-Modena, William M. de Souza

**Affiliations:** University of Kentucky, Lexington, Kentucky, USA (S.T.S. de Lima, I.M. Claro, X. Hua, C. McDonald, W.M. de Souza); Universidade Estadual de Campinas, Campinas, Brazil (S.T.S. de Lima, L. Sacchetto, J. Forato, J.L. Proenca-Modena); Laboratório Central de Saúde Pública do Ceará, Fortaleza, Brazil (S.T.S. de Lima, C.P.M. Ferraz, L.M.F. Duarte, V.A.F. Viana, A.C. Firmino, D.B. Silva, S.M.S. Macedo); Universidade Federal de Minas Gerais, Belo Horizonte, Brazil (R. de Jesus, L.M. Simões Mello); Universidade de São Paulo, São Paulo, Brazil (E.R. Manuli, E.C. Sabino); Universidade Municipal de São Caetano do Sul, São Caetano do Sul, Brazil (E.R. Manuli); Universidade de Fortaleza, Fortaleza (M.E.T. de Lima); Laboratório Central de Saúde Pública do Acre, Rio Branco, Brazil (M.A. Costa); Laboratório Central de Saúde Pública do Rio Grande do Norte, Natal, Brazil (J.J.P.A. Abrantes); Laboratório Central de Saúde Pública do Sergipe, Aracaju, Brazil (L.O.C. Sena); Universidade Federal do Vale do São Francisco, Petrolina, Brazil (R.F. do Carmo); FIOCRUZ, Campo Grande, Brazil (J. Croda)

**Keywords:** dengue, viruses, vector-borne infections, Brazil

## Abstract

We evaluated the transmission dynamics of dengue virus (DENV) during 2010–2025 in Ceará State, Brazil. Ceará has been a dengue hyperendemic region, where all 4 DENV serotypes co-circulate, predominantly DENV-1 and DENV-2, and where DENV-3 was recently reintroduced. Our findings provide an epidemiologic baseline for postlicensure dengue vaccine evaluation.

Dengue virus (DENV) is an arbovirus estimated to cause >390 million infections annually worldwide, leading to >10,000 deaths in >125 countries (*1*,*2*). DENV (Flaviviridae family) is transmitted to humans primarily by *Aedes aegypti* and *Ae. albopictus* mosquitoes (*3*) and comprises 4 genetically and antigenically distinct serotypes (DENV-1–4) (*4*). DENV infection can be categorized as dengue without warning signs, dengue with warning signs, and severe dengue, which can include death (*5*). No specific antiviral treatment is available for dengue. As of August 2026, Dengvaxia (Sanofi Pasteur, https://www.sanofi.us), Qdenga TAK-003 (Takeda, https://www.takeda.com), and Butantan-DV (Instituto Butantan, https://en.butantan.gov.br) are 3 licensed vaccines to prevent dengue; however, Dengvaxia use remains limited because of safety concerns (*6*,*7*). Brazil remains one of the most affected countries by dengue and has annual epidemics, widespread vector distribution, and co-circulation of all 4 DENV serotypes (*8*). We integrate epidemiologic and genomic methods to investigate DENV transmission dynamics during 2010–2025 in Ceará, a hyperendemic state in Brazil.

## The Study

We retroactively obtained national epidemiologic data on laboratory-confirmed dengue cases during January 2010–January 2025 from Brazil’s Ministry of Health. We obtained additional epidemiologic data from the Central Laboratory of Public Health of Ceará for dengue cases reported during July 2014–June 2025. We used the MiSeq platform (Illumina, https://www.illumina.com) to sequence residual quantitative reverse transcription PCR–positive DENV serum samples collected from case-patients with DENV-1–3 serotypes during February 2024–July 2025. We assigned DENV lineages by using the DENV typing tool (*9*), followed by phylogenetic reconstruction. We also performed time-series, spatial mapping, and demographic analyses (Appendix).

During January 3, 2010–January 25, 2025, Brazil reported 1,657,046 laboratory-confirmed dengue cases across 98.9% (5,509/5,570) of municipalities in all 26 states and the Federal District. We identified 13 major epidemic waves at the national level; annual incidence varied from 4.0 to 190.4 cases/100,000 inhabitants, and the largest epidemic occurred in 2024 (Figure 1, panel A). National DENV testing positivity rate ranged from 18.2% (19,460/106,901 persons tested) to 57.1% (146,649/256,981 persons tested) during those 15 years (Figure 1, panel B). In Ceará, a total of 131,961 laboratory-confirmed cases were reported, corresponding to a cumulative incidence of 1,500 cases/100,000 inhabitants. Ceará epidemic peaks coincided with national trends but frequently reached higher incidence and positivity rates (Figure 1). 

**Figure 1:**
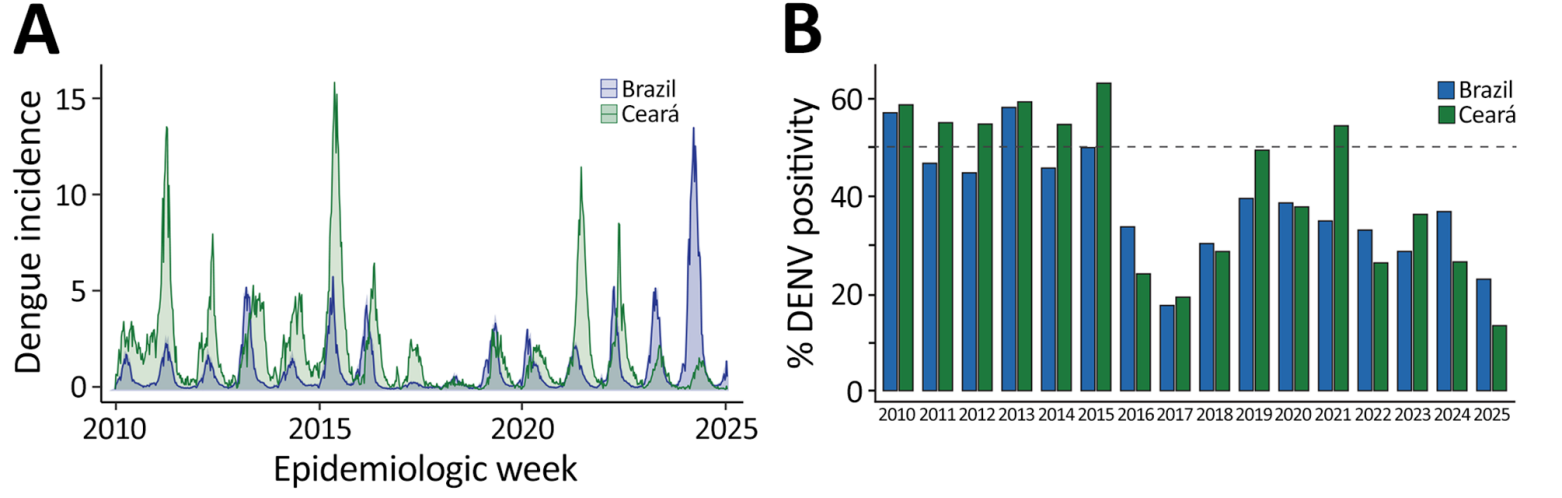
Dengue incidence and DENV test positivity from study of DENV transmission dynamics, Ceará State, Brazil, 2010–2025. A) Weekly incidence of laboratory-confirmed dengue cases/100,000 inhabitants at the national (Brazil) and state (Ceará) levels, from epidemiologic week 1 of 2010 (January 3–9) through epidemiologic week 4 of 2025 (January 20–26). B) Annual DENV test positivity rate (%) in Brazil and Ceará. Dashed line indicates 50% positivity. DENV, dengue virus.

By 2026, dengue had been reported in all 184 municipalities in Ceará; cumulative incidences range from 41.4 to 8,566 cases/100,000 inhabitants (Appendix). Nationally, dengue epidemics occurred mainly during February–June and peaked during March–April (peak monthly incidence mean 10.4 cases/100,000 inhabitants). In contrast, in Ceará, epidemics occurred during March–August, typically peaking in May (incidence mean 18.4 cases/100,000 inhabitants) (Appendix). Sex- and age-stratified analyses revealed a female-to-male ratio of 1.6 and higher incidence among female persons nationally (2-way ANOVA test, p<0.05) and in Ceará (p<0.01) (Appendix). The cumulative incidence in Ceará was higher in persons 10–39 years of age than in persons >70 years of age (p<0.05) (Appendix).

We investigated the circulation of DENV serotypes in Ceará during July 2014–June 2025. DENV-1 was the predominant DENV serotype during 2014–2019 and again during 2023–2025 (Figures 2, 3). Fortaleza, the capital and most populous city in Ceará, reported co-circulation of DENV-1, DENV-3, and DENV-4 in 2015. DENV-2 reemerged in 2019, leading to its near-complete replacement of other DENV serotypes in 2021 and 2022 (Figures 2, 3). To assess current genetic diversity in Ceará, we sequenced 243 DENV genomes from cases reported during February 2024–July 2025 across 45 municipalities (Figure 4; Appendix). Cycle threshold values ranged from 10.6 to 33.0. We recovered complete or partial DENV genomes for 218 of the 243 samples (coverage 14.4%–99.2%; mean depth 16–9,877 per nucleotide) (Appendix). Among those genomes, we classified 82.6% (180/218) with >70% coverage into the following genotypes: DENV-1 V_D1.1 (n = 1), DENV-1 V_E1 (n = 111), DENV-2 II_F1.1.2 (n = 61), DENV-2 II_F1.1.5 (n = 2), DENV-2 III_C1.1 (n = 2), and DENV-3 III_B3.2 (n = 3) (*9*). We included those newly generated DENV genome sequences in maximum-likelihood phylogenetic analyses along with global DENV genome references (Appendix).

**Figure 2:**
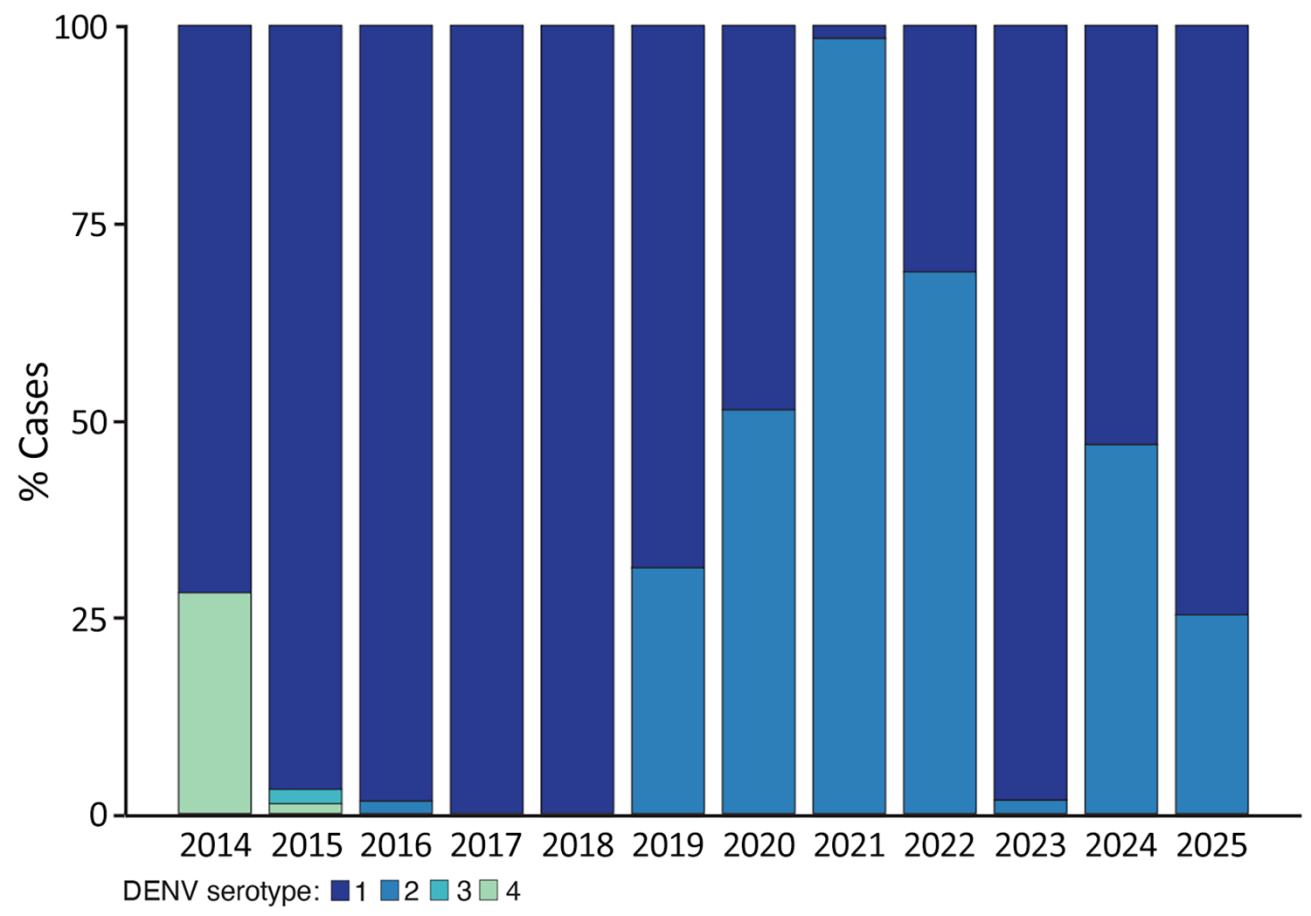
Annual percentage of dengue cases attributed to DENV serotypes 1, 2, 3, or 4 from study of DENV transmission dynamics, Ceará State, Brazil, 2014–2025. DENV, dengue virus.

**Figure 3:**
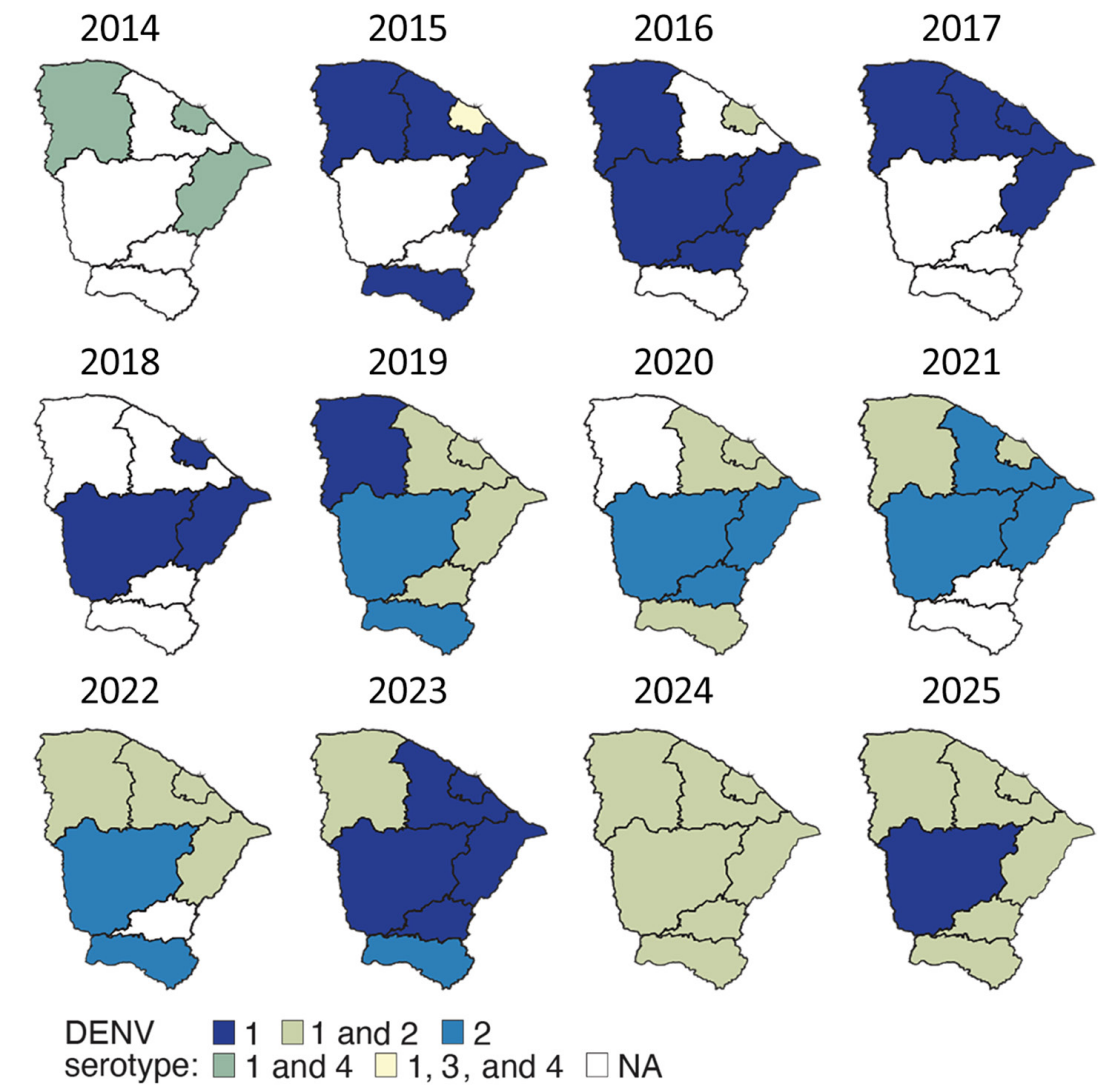
Annual distribution of DENV serotypes across the mesoregions from study of DENV transmission dynamics, Ceará State, Brazil, 2014–2025. DENV, dengue virus.

**Figure 4:**
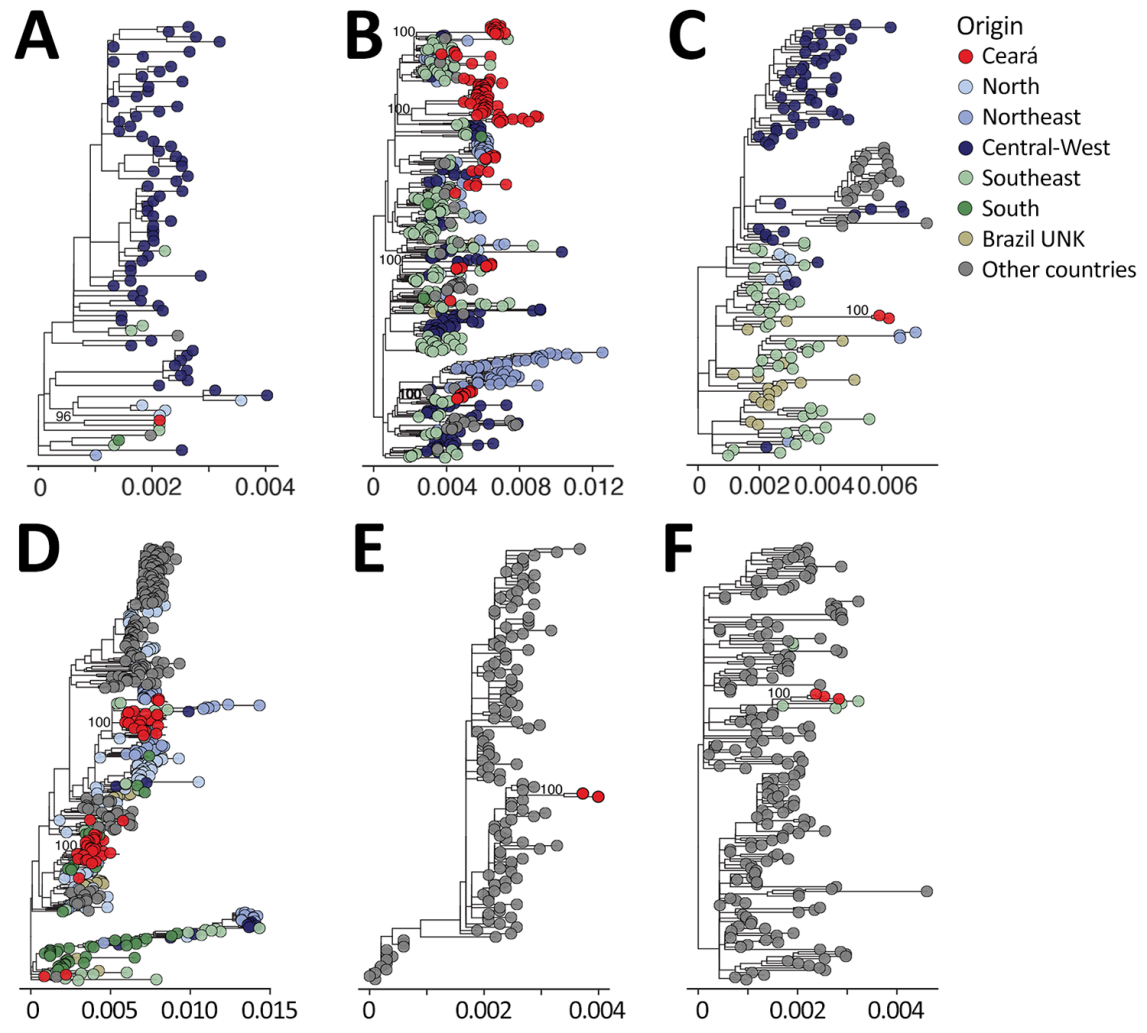
Maximum-likelihood phylogenetic trees of DENV lineages detected from study of DENV transmission dynamics, Ceará State, Brazil, February 2024–July 2025. A) DENV-1 lineage V_D1.1; B) DENV-1 lineage V_E1; C) DENV-2 lineage III_C1.1; D) DENV-2 lineage II_F1.1.2; E) DENV-2 lineage II_F1.1.5; F) DENV-3 lineage III_B3.2. A total of 180 DENV genomes generated in this study from Ceará were analyzed alongside publicly available global references. Final datasets comprised DENV-1 lineage V_D1.1 (n = 218), DENV-1 lineage V_E1 (n = 791), DENV-2 lineage II_F1.1.2 (n = 499), DENV-2 lineage II_F1.1.5 (n = 133), DENV-2 lineage III_C1.1 (n = 366), and DENV-3 lineage III_B3.2 (n = 734). Tips are colored by region or state of origin. Red dots indicate newly generated DENV sequences in this study. Phylogenies are midpoint-rooted for clarity. Scale bars represent substitutions per nucleotide site. Bootstrap support values (1,000 replicates) are shown at key nodes. Complete phylogenies for each lineage and GenBank accession numbers for all sequences used are provided (Appendix). DENV, dengue virus; UNK, unknown region of Brazil.

Phylogenetic analysis showed that the recent epidemic in Ceará (2024–2025) was mainly caused by DENV-1 lineage V_E1 (61.6% [n = 111 cases]). DENV lineages identified in Ceará were distributed across multiple well-supported subclades with genomes from southeast (São Paulo), central-west (Federal District, Goiás, and Mato Grosso do Sul), and northeast (Alagoas) of Brazil and from Florida (USA) and Uruguay. Lineage II_F1.1.2 (94%) predominated among DENV-2 cases, and clustered with DENV genomes from southeast and northeast Brazil and the United States (*10*). Conversely, DENV-2 II_F1.1.5 genomes grouped with sequences from France (2023–2024) and the Dominican Republic (2024), suggesting a recent international introduction. Detection of DENV-3 genomes from Ceará was consistent with a reintroduction because no local detections have occurred since 2015. Those genomes belonged to lineage III_B3.2 and formed a well-supported clade (bootstrap 100%) with sequences from São Paulo (2024–2025) and Florida (2023) within a lineage widely circulating across the Americas, including in Cuba, the Dominican Republic, French Guiana, and Peru (Figure 4).

This study provides a 15-year overview of dengue transmission in Ceará, a dengue hyperendemic state. Our epidemiologic analyses demonstrate that dengue occurred in all 184 municipalities of Ceará and that multiple DENV epidemics reached higher incidence and testing positivity rates than national trends. Epidemic peaks typically occurred ≈1 month later and lasted longer in Ceará than the national average, which might be explained by extended periods of high temperature and humidity (*11*), supporting near year-round vector abundance and sustained DENV transmission (*3*).

We identified heterogeneous circulation of all 4 DENV serotypes and identified 6 distinct viral lineages across 3 serotypes (DENV-1, DENV-2, and DENV-3) sustained by multiple national and international introductions. We also documented the reintroduction of DENV-3, confirmed by its detection in July 2025, which could increase the risk for severe outcomes in the coming years through sequential infections and antibody-dependent enhancement after a decade of co-circulation of DENV-1 and DENV-2 (*12*). Moreover, our phylogenetic analyses showed that DENV-3 lineage III_B3.2 genomes clustered with DENV sequences detected during 2024–2025 in São Paulo State. That finding suggests a recent introduction of DENV-3 into Ceará through domestic routes, after its dissemination and sustained circulation across different regions of Brazil, rather than importation from the Caribbean and the Americas (*13*,*14*).

One limitation of our study is its reliance on variable healthcare-seeking behaviors, which might not capture the full extent of dengue cases, including asymptomatic cases. In addition, on the basis of our epidemiological data, we cannot distinguish between primary and secondary DENV infections. Moreover, we acknowledge that some dengue cases diagnosed by IgM detection might reflect cross-reactivity with Zika virus, which emerged in Brazil in 2015 (*15*). Furthermore, our genomic data are limited to 2024–2025, which, together with the scarcity of DENV genomic data from Ceará in public genomic databases, prevents elucidation of the spatial and temporal dynamics of DENV lineages in Ceará before 2024.

## Conclusions

In summary, our study described the long-term dengue transmission dynamics in Ceará, Brazil, during 2010–2025 and substantially increased the DENV genomic data from this hyperendemic region. Such genomic data enable identification of circulating and emerging DENV lineages, elucidating the spatiotemporal evolution and shifts in DENV patterns. Our findings will guide public health management and future efforts to implement dengue immunization programs using Qdenga TAK-003 and Butantan-DV in Brazil. Co-circulation of all DENV serotypes, and the current predominance of DENV-1 and DENV-2, highlight the necessity for continued serotype-specific surveillance to further monitor vaccine effectiveness. 

Appendix Additional information about transmission dynamics of dengue virus, Ceará State, Brazil, 2010–2025.
