# Supplementary material for: Transmission Dynamics of Dengue Virus, Ceará State, Brazil, 2010–2025
Source: Emerg Infect Dis. 2026 Nov;32(11):1897–901. doi: 10.3201/eid3211.260609 (PMC13715876; doi:10.3201/eid3211.260609)
Supplement: Supplementary file 1 [file 26-0609-Techapp-s1.pdf]

Article DOI: https://doi.org/10.3201/eid3211.260609

EID cannot ensure accessibility for supplementary materials supplied by authors. Readers who have difficulty accessing supplementary content should contact the authors for assistance.

Transmission Dynamics of Dengue Virus, Ceará State, Brazil, 2010–2025

Appendix

Materials and Methods

Definition of cases

Epidemiological data on laboratory-confirmed dengue cases from epidemiological week 1 of 2010 (January 3–9) through week 4 of 2025 (January 20–26) were obtained from the Brazilian Ministry of Health. This dataset included patient demographics (i.e., age and sex), dates of symptom onset and sample collection, laboratory diagnostic results, and dengue virus (DENV) serotype. Laboratory-confirmed dengue cases were defined as patients with a positive result by at least one of the following diagnostic methods: reverse-transcription quantitative polymerase chain reaction (RT-qPCR), immunoglobulin M (IgM) detection, and/or non-structural protein 1 (NS1) antigen detection. Dengue cases based solely on clinical suspicion were not included in this study.

Real-time quantitative reverse transcription-polymerase chain reaction (RT-qPCR) for dengue virus

Viral RNA was extracted from the serum samples using the Maxwell HT Viral TNA Kit (Cat no. AX2340, Promega, USA) with the KingFisher Flex Purification System robot (Thermo Fisher Scientific, USA), following the manufacturer's instructions. Extracted RNA was tested for DENV serotypes 1–4 (*1*,*2*) (Appendix Table 1) with the *Instituto de Biologia Molecular do Paraná* (IBMP) Zika, Dengue, and Chikungunya kit (Fiocruz, Brazil). Reactions were performed on the QuantStudio 3 platform (Applied Biosystems, USA). Results were considered positive if RT-qPCR cycle quantification threshold (Ct) values were less than 36 according to manufacturers.

Epidemiological and statistical analysis

Incidence rates were calculated using the 2022 Brazilian population census (IBGE, www.ibge.gov.br). Comparisons of sex and age groups were conducted using one-way ANOVA and Tukey's Honest Significant Difference (HSD) test. Differences were considered statistically significant if $p < 0.05$. Analyses were performed in RStudio version 4.4.0 (https://posit.co).

Dengue virus genome sequencing and analysis

Near-complete genome sequencing was performed on DENV-positive samples identified by RT-qPCR from February 4, 2024, and July 8, 2025 (Appendix Table 2), using a multiplex PCR scheme previously described for DENV serotypes 1–4 (*3*). Libraries were prepared using the Illumina COVIDSeq Test (Illumina, USA) and sequenced on an Illumina MiSeq System (V2 cartridge, 300 bp cycles) (Illumina, USA) following the manufacturer's instructions. Raw FASTQ files were trimmed with Trimmomatic v0.39 (*4*) (i.e., low-quality reads, minimum Phred score, short reads, primer removal), aligned to reference genomes (GenBank accessions NC_001477.1, NC_001474.2, NC_001475.2 for DENV-1, DENV-2, DENV-3, respectively) with Minimap2 v2.22 (*5*), and processed with SAMtools v1.11 (*6*). Variants and consensus sequences were generated with BCFtools v1.11 (*7*). Regions with <50× coverage were masked as "N". Genome statistics were computed using Bedtools v2.30.0 (*8*) and SAMtools. Summary assembly statistics are presented in Appendix Table 3.

Phylogenetic analysis

Consensus sequences were aligned with publicly available DENV genomes (≥70% coverage; 7,139 DENV-1, 6,296 DENV-2, 3,132 DENV-3 sequences from NCBI Virus, accessed July 25, 2025) using MAFFT version 7.525 (*9*). Alignments were manually refined using AliView version 1.2 (*10*). Maximum likelihood (ML) phylogenies were inferred using IQ-TREE version 2 under the GTR+F+I+G4 model, as determined by ModelFinder (*11*,*12*). Branch support was assessed using 1,000 ultrafast bootstrap replicates. DENV typing tool was used to identify genotype and lineage placement of each sample (*13*,*14*). Subsets were then generated for each lineage clade identified: DENV-11 lineage V_D1.1 (n=218), DENV-1 lineage V_E1 (n=791), DENV-2 lineage II_F1.1.2 (n=499), DENV-2 lineage II_F1.1.5 (n=133), DENV-2 lineage III_C1.1 (n=366), and DENV-3 lineage III_B3.2 (n=734). Trees were visualized in FigTree version 1.4.4 (http://tree.bio.ed.ac.uk/software/figtree/).

References

1. Johnson BW, Russell BJ, Lanciotti RS. Serotype-specific detection of dengue viruses in a fourplex real-time reverse transcriptase PCR assay. J Clin Microbiol. 2005;43:4977–83. PubMed https://doi.org/10.1128/JCM.43.10.4977-4983.2005

2. Callahan JD, Wu SJ, Dion-Schultz A, Mangold BE, Peruski LF, Watts DM, et al. Development and evaluation of serotype- and group-specific fluorogenic reverse transcriptase PCR (TaqMan) assays for dengue virus. J Clin Microbiol. 2001;39:4119–24. PubMed https://doi.org/10.1128/JCM.39.11.4119-4124.2001

3. Quick J, Grubaugh ND, Pullan ST, Claro IM, Smith AD, Gangavarapu K, et al. Multiplex PCR method for MinION and Illumina sequencing of Zika and other virus genomes directly from clinical samples. Nat Protoc. 2017;12:1261–76. PubMed https://doi.org/10.1038/nprot.2017.066

4. Bolger AM, Lohse M, Usadel B. Trimmomatic: a flexible trimmer for Illumina sequence data. Bioinformatics. 2014;30:2114–20. PubMed https://doi.org/10.1093/bioinformatics/btu170

5. Li H. Minimap2: pairwise alignment for nucleotide sequences. Bioinformatics. 2018;34:3094–100. PubMed https://doi.org/10.1093/bioinformatics/bty191

6. Li H, Handsaker B, Wysoker A, Fennell T, Ruan J, Homer N, et al.; 1000 Genome Project Data Processing Subgroup. The Sequence Alignment/Map format and SAMtools. Bioinformatics. 2009;25:2078–9. PubMed https://doi.org/10.1093/bioinformatics/btp352

7. Danecek P, Bonfield JK, Liddle J, Marshall J, Ohan V, Pollard MO, et al. Twelve years of SAMtools and BCFtools. Gigascience. 2021;10:giab008. PubMed https://doi.org/10.1093/gigascience/giab008

8. Quinlan AR, Hall IM. BEDTools: a flexible suite of utilities for comparing genomic features. Bioinformatics. 2010;26:841–2. PubMed https://doi.org/10.1093/bioinformatics/btq033

9. Katoh K, Standley DM. MAFFT multiple sequence alignment software version 7: improvements in performance and usability. Mol Biol Evol. 2013;30:772–80. PubMed https://doi.org/10.1093/molbev/mst010

10. Larsson A. AliView: a fast and lightweight alignment viewer and editor for large datasets. Bioinformatics. 2014;30:3276–8. PubMed https://doi.org/10.1093/bioinformatics/btu531

11. Kalyaanamoorthy S, Minh BQ, Wong TKF, von Haeseler A, Jermiin LS. ModelFinder: fast model selection for accurate phylogenetic estimates. Nat Methods. 2017;14:587–9. PubMed https://doi.org/10.1038/nmeth.4285

12. Minh BQ, Schmidt HA, Chernomor O, Schrempf D, Woodhams MD, von Haeseler A, et al. IQ-TREE 2: new models and efficient methods for phylogenetic inference in the genomic era. Mol Biol Evol. 2020;37:1530–4. PubMed https://doi.org/10.1093/molbev/msaa015

13. Fonseca V, Libin PJK, Theys K, Faria NR, Nunes MRT, Restovic MI, et al. A computational method for the identification of dengue, Zika and chikungunya virus species and genotypes. PLoS Negl Trop Dis. 2019;13:e0007231. PubMed https://doi.org/10.1371/journal.pntd.0007231

14. Hill V, Cleemput S, Pereira JS, Gifford RJ, Fonseca V, Tegally H, et al. A new lineage nomenclature to aid genomic surveillance of dengue virus. PLoS Biol. 2024;22:e3002834. PubMed https://doi.org/10.1371/journal.pbio.3002834

Appendix Table 1. Primers and probes used for the detection of arboviruses in this study

Virus	Sequences (5'→3')	Primers and probes orientation	Target	Genome position*	Ref.
DENV1	GACACCACACCCTTTGGACAA	Forward	NS5	8586-8606	(*2*)
	CACYTGGGCTGTCACCTCCAT	Reverse	NS5	8692-8673	
	AGAGGGTGTTTAAAGAGAAAGTTGACACGCG	Probe		8608-8638	
DENV2	CAGGTTATGGCACTGTCACGAT	Forward	M	1605	(*1*)
	CCATCTGCAGCAACACCATCTC	Reverse	M	1583	
	CTCCGAGAACAGGCCTCGACTTCAA	Probe		1008	
DENV3	GGGAAAACCGTCTATCAATA	Forward	C	118-221	(*2*)
	CGCCATAACCAATTTCATTGG	Reverse	C	241-221	
	CACAGTTGGCGAAGAGATCTCAAGAGGA	Probe		174-202	
DENV4	TGAAGAGATTCTCAACCGGAC	Forward	C	187-207	(*2*)
	AATCCCTGCTGTTGGTGGC	Reverse	C	293-275	
	TCATCACGTTTTTGCGAGTCCTTTCCA	Probe		247-273	

*Positions are indicated relative to the reference sequences (GenBank accessions NC_001477.1, NC_001474.2, NC_001475.2 for DENV-1, DENV-2, DENV-3 respectively). Legend: DENV, dengue virus. NS5, non-structural protein 5. M, matrix protein. C, capsid protein.

Appendix Table 2. Information on samples of patients RT-qPCR-positive for DENV-1-3 sequenced in this study (n=243)

Sample ID	Serotype	Onset symptoms	Collection date	Sex	Age	Municipality
SEQ03_24_13	DENV1	2024-03-25	26/03/2024	F	19	Barroquinha
SEQ03_24_07	DENV1	2024-03-29	31/03/2024	M	26	Jaguaribe
SEQ03_24_01	DENV1	2024-02-23	25/02/2024	F	22	Jaguaribe
SEQ03_24_04	DENV1	2024-03-07	2024-11-03	M	30	Cruz
SEQ03_24_03	DENV1	2024-03-03	2024-06-03	F	29	Cruz
SEQ03_24_09	DENV1	2024-03-29	2024-02-04	F	12	Fortaleza
SEQ03_24_12	DENV1	2024-03-25	26/03/2024	F	52	Barroquinha
SEQ03_24_15	DENV1	2024-03-31	2024-02-04	M	32	Cruz
SEQ03_24_05	DENV1	2024-03-11	13/03/2024	F	29	Pedra Branca
SEQ03_24_06	DENV1	2024-03-19	21/03/2024	F	16	Fortaleza
SEQ03_24_08	DENV1	2024-04-07	2024-08-04	F	20	Barroquinha
SEQ03_24_10	DENV1	2024-04-13	15/04/2024	M	27	Limoeiro Do Norte
SEQ03_24_11	DENV1	2024-03-19	20/03/2024	F	53	Jaguaribe
SEQ03_24_14	DENV1	2024-03-31	2024-03-04	M	12	Barroquinha
SEQ03_24_16	DENV1	2024-04-01	2024-04-04	M	18	Barroquinha
SEQ03_24_17	DENV1	2024-04-07	2024-09-04	F	12	Taua
SEQ03_24_18	DENV1	2024-04-06	2024-10-04	F	61	Cruz
SEQ03_24_20	DENV1	2024-04-21	23/04/2024	M	16	Barroquinha
SEQ03_24_02	DENV1	2024-03-02	2024-03-03	F	57	Fortaleza
SEQ03_24_19	DENV1	2024-04-16	18/04/2024	F	6	Fortaleza
SEQ08_25_31	DENV2	Unk	2025-02-21	F	55	Barbalha
SEQ05_24_12	DENV2	2024-04-28	2024-04-30	M	18	Barroquinha
SEQ05_24_11	DENV2	2024-04-30	2024-05-01	M	15	Barroquinha
SEQ05_24_40	DENV2	2024-06-04	2024-06-06	M	40	Cruz

Sample ID	Serotype	Onset symptoms	Collection date	Sex	Age	Municipality
SEQ05_25_36	DENV2	2024-12-24	2024-12-29	F	47	Penaforte
SEQ13_25_15	DENV1	2025-05-11	2025-05-16	M	32	Milagres
SEQ05_24_20	DENV2	2024-05-04	2024-05-07	F	14	Sao Benedito
SEQ13_25_14	DENV1	2025-05-17	2025-05-20	M	68	Jati
SEQ05_24_21	DENV2	2024-05-04	2024-05-08	F	25	Fortaleza
SEQ05_24_14	DENV2	2024-05-01	2024-05-02	F	41	Jaguaribe
SEQ09_25_27	DENV2	2025-05-11	2025-05-14	M	21	Russas
SEQ05_24_31	DENV1	2024-05-18	2024-05-22	M	20	Fortaleza
SEQ05_24_39	DENV2	2024-05-25	2024-05-28	F	50	Taua
SEQ08_25_05	DENV1	2025-01-14	2025-01-17	M	18	Redencao
SEQ08_25_27	DENV2	Unk	2025-02-21	M	2	Penaforte
SEQ09_25_22	DENV2	2025-04-29	2025-05-02	F	20	Cruz
SEQ08_25_07	DENV1	2025-01-26	2025-01-31	M	30	Redencao
SEQ08_25_32	DENV2	2025-01-10	2025-01-18	F	44	Penaforte
SEQ08_25_25	DENV2	2025-01-10	2025-01-11	M	4	Penaforte
SEQ08_25_04	DENV1	2025-03-15	2025-03-18	M	55	Redencao
SEQ05_25_02	DENV2	2024-04-28	2024-04-28	M	18	Barroquinha
SEQ08_25_18	DENV1	2025-03-08	2025-03-13	M	39	Redencao
SEQ05_24_24	DENV1	2024-05-01	2024-05-01	M	28	Carnaubal
SEQ08_25_24	DENV2	2024-12-26	2024-12-30	M	39	Pontal
SEQ09_25_26	DENV2	2025-05-05	2025-05-13	M	27	Tiangua
SEQ05_24_19	DENV2	2024-05-08	2024-05-09	M	9	Barroquinha
SEQ13_25_46	DENV3	Unk	2025-07-02	M	28	Barbalha
SEQ05_25_39	DENV1	2025-01-26	2025-01-31	F	30	Redencao
SEQ09_24_10	DENV2	2024-06-24	2024-06-28	F	29	Fortaleza
SEQ08_25_06	DENV1	2025-01-26	2025-01-31	M	24	Redencao
SEQ13_25_43	DENV2	2025-06-08	2025-06-10	F	73	Santana Do Cariri
SEQ09_24_14	DENV1	2024-07-11	2024-07-15	M	33	Fortim
SEQ09_25_25	DENV2	2025-05-03	2025-05-07	F	56	Fortaleza
SEQ05_25_11	DENV2	2024-05-04	2024-05-08	F	25	Brasilia
SEQ09_24_08	DENV2	2024-06-23	2024-06-26	M	43	Cruz
SEQ05_24_26	DENV2	2024-05-12	2024-05-15	F	14	Fortaleza
SEQ13_25_40	DENV2	2025-06-02	2025-06-04	M	24	Missao Velha
SEQ09_25_21	DENV2	2025-04-15	2025-04-17	F	47	Fortaleza
SEQ09_24_28	DENV1	2024-08-23	2024-08-30	M	46	Potiretama
SEQ09_25_19	DENV2	Unk	2025-03-21	M	43	Jardim
SEQ09_24_05	DENV2	2024-06-19	2024-06-18	F	6	Cruz
SEQ08_25_12	DENV1	2025-03-02	2025-03-10	F	25	Redencao
SEQ09_25_20	DENV2	Unk	2025-03-20	F	12	Barbalha
SEQ13_25_47	DENV3	Unk	2025-07-08	M	8	Barbalha
SEQ05_24_23	DENV1	2024-05-12	2024-05-13	M	27	Caninde
SEQ13_25_36	DENV2	2025-05-17	2025-05-19	M	17	Abaiara
SEQ05_24_25	DENV1	2024-05-13	2024-05-16	F	44	Fortaleza
SEQ09_24_29	DENV1	2024-09-09	2024-09-12	F	60	Quixere
SEQ09_25_15	DENV1	2025-05-16	2025-05-20	M	39	Fortaleza
SEQ08_25_10	DENV1	2025-02-22	2025-02-27	F	51	Redencao
SEQ08_25_35	DENV2	2025-02-04	2025-02-08	M	17	Fortaleza
SEQ08_25_30	DENV2	Unk	2025-02-21	M	43	Barbalha
SEQ13_25_34	DENV2	2025-05-20	2025-05-23	M	33	Jijoca De Jericoacoara
SEQ09_25_23	DENV2	2025-04-25	2025-04-27	M	33	Sao Carlos
SEQ09_24_25	DENV2	2024-07-30	2024-08-12	F	50	Maracanau
SEQ09_24_01	DENV1	2024-06-06	2024-06-10	F	48	Brejo Santo
SEQ09_24_22	DENV1	2024-08-01	2024-08-02	F	30	Graca
SEQ13_25_24	DENV1	2025-06-13	2025-06-16	M	52	Senador Pompeu
SEQ09_25_24	DENV2	2025-05-01	2025-05-06	M	27	Tiangua
SEQ13_25_30	DENV1	2025-06-07	2025-06-09	M	16	Redencao
SEQ05_24_35	DENV1	2024-05-31	2024-06-02	F	17	Fortim
SEQ09_24_17	DENV1	2024-07-21	2024-07-23	M	28	Fortim
SEQ08_25_33	DENV2	Unk	2025-02-24	M	25	Penaforte
SEQ05_24_37	DENV2	2024-05-28	2024-05-29	F	64	Fortim
SEQ08_25_28	DENV2	Unk	2025-02-21	F	55	Penaforte
SEQ05_24_36	DENV2	2024-05-26	2024-05-29	F	18	Cruz
SEQ05_24_15	DENV2	2024-05-01	2024-05-02	M	33	Graca
SEQ05_24_28	DENV2	2024-05-20	2024-05-21	F	26	Barroquinha
SEQ05_24_29	DENV2	2024-05-18	2024-05-22	M	47	Jaguaribe
SEQ13_25_41	DENV2	2025-06-11	2025-06-13	F	62	Juazeiro Do Norte
SEQ05_24_18	DENV2	2024-05-06	2024-05-09	F	34	Graca
SEQ09_25_14	DENV1	Unk	2025-03-21	F	38	Milagres
SEQ05_25_06	DENV2	2024-05-01	2024-05-02	F	41	Jaguaribe

Sample ID	Serotype	Onset symptoms	Collection date	Sex	Age	Municipality
SEQ05_24_30	DENV2	2024-05-23	2024-05-27	F	51	Taua
SEQ05_25_35	DENV2	2025-01-14	2025-01-14	M	20	Penaforte
SEQ05_24_34	DENV1	2024-05-28	2024-05-29	F	37	Fortim
SEQ13_25_45	DENV3	2025-06-02	2025-06-06	F	9	Barbalha
SEQ05_25_01	DENV2	2024-04-30	2024-05-01	M	15	Barroquinha
SEQ09_24_11	DENV1	2024-07-03	2024-07-04	F	61	Fortaleza
SEQ09_24_27	DENV1	2024-08-26	2024-08-27	F	21	Potiretama
SEQ09_24_30	DENV1	Unk	2024-09-18	F	14	Taua
SEQ09_24_16	DENV1	2024-07-19	2024-07-23	M	14	Fortim
SEQ09_24_03	DENV2	2024-06-15	2024-06-17	F	34	Caucaia
SEQ05_25_38	DENV1	2025-01-26	2025-01-31	M	24	Redencao
SEQ08_25_47	DENV1	2025-03-31	2025-04-02	M	8	Acarape
SEQ08_25_40	DENV2	Unk	2025-02-12	M	4	Penaforte
SEQ09_24_19	DENV1	2024-07-25	2024-07-29	M	18	Fortim
SEQ09_24_23	DENV1	2024-08-06	2024-08-05	F	21	Icapui
SEQ05_25_29	DENV2	2024-06-04	2024-06-05	M	40	Cruz
SEQ08_25_38	DENV2	Unk	2025-02-06	F	55	Penaforte
SEQ08_25_19	DENV1	2025-03-10	2025-03-14	F	39	Redencao
SEQ08_25_21	DENV1	Unk	2025-03-10	M	57	Jardim
SEQ05_25_16	DENV2	2024-05-12	2024-05-16	F	14	Sao Goncalo Do Amarante
SEQ08_25_01	DENV1	2025-01-05	2025-01-08	F	15	Acarape
SEQ08_25_09	DENV1	2025-02-14	2025-02-20	M	19	Redencao
SEQ08_25_22	DENV1	Unk	2025-03-10	M	1	Barbalha
SEQ08_25_14	DENV1	2025-03-08	2025-03-11	F	29	Redencao
SEQ08_25_46	DENV1	2025-03-23	2025-03-26	F	11	Acarape
SEQ09_25_09	DENV1	2025-04-28	2025-04-28	F	20	Acarape
SEQ13_25_03	DENV1	2025-05-11	2025-05-15	F	11	Jardim
SEQ09_24_09	DENV1	2024-06-25	2024-06-26	F	1	Fortaleza
SEQ13_25_28	DENV1	2025-06-06	2025-06-10	F	11	Barbalha
SEQ09_25_17	DENV1	Unk	2025-03-21	M	43	Jardim
SEQ09_24_24	DENV1	2024-08-09	2024-08-08	F	26	Jardim
SEQ09_25_18	DENV2	Unk	2025-03-21	M	24	Jardim
SEQ09_24_07	DENV1	2024-06-15	2024-06-21	F	22	Cruz
SEQ09_24_18	DENV1	2024-07-26	2024-07-29	F	39	Fortim
SEQ05_25_32	DENV2	2025-01-08	2025-01-10	F	66	Penaforte
SEQ13_25_37	DENV2	2025-05-23	2025-05-26	M	12	Penaforte
SEQ08_25_36	DENV2	Unk	2025-02-06	F	22	Penaforte
SEQ08_25_13	DENV1	2025-03-07	2025-03-11	F	51	Redencao
SEQ09_25_07	DENV1	2025-04-24	2025-04-28	F	25	Pacoti
SEQ08_25_15	DENV1	2025-03-11	2025-03-12	M	47	Baturite
SEQ13_25_08	DENV1	2025-05-16	2025-05-20	M	33	Oros
SEQ08_25_17	DENV1	2025-03-10	2025-03-13	M	8	Redencao
SEQ08_25_26	DENV2	Unk	2025-02-21	F	13	Penaforte
SEQ09_25_04	DENV1	Unk	2025-03-20	F	7	Orós
SEQ08_25_20	DENV1	2025-03-10	2025-03-14	F	28	Redencao
SEQ09_25_11	DENV1	2025-04-25	2025-04-29	M	19	Redencao
SEQ05_25_22	DENV1	2024-05-23	2024-05-28	F	62	Fortaleza
SEQ09_24_06	DENV1	2024-06-15	2024-06-20	M	54	Cruz
SEQ09_24_12	DENV1	2024-07-02	2024-07-06	F	25	Fortaleza
SEQ13_25_35	DENV2	2025-05-22	2025-05-23	F	32	Jijoca De Jericoacoara
SEQ05_24_38	DENV1	2024-05-21	2024-05-22	F	37	Brejo Santo
SEQ08_25_29	DENV2	Unk	2025-02-21	M	45	Penaforte
SEQ08_25_41	DENV2	Unk	2025-02-12	M	60	Penaforte
SEQ13_25_38	DENV2	2025-05-22	2025-05-26	M	18	Penaforte
SEQ13_25_39	DENV2	2025-06-03	2025-06-03	M	1	Santana Do Cariri
SEQ08_25_34	DENV2	Unk	2025-02-24	F	5	Penaforte
SEQ08_25_43	DENV2	Unk	2025-02-12	M	47	Penaforte
SEQ09_24_13	DENV1	2024-07-04	2024-07-08	M	49	Fortim
SEQ13_25_33	DENV2	2025-05-19	2025-05-19	M	19	Crato
SEQ08_25_03	DENV1	2025-01-09	2025-01-11	F	33	Penaforte
SEQ08_25_39	DENV2	Unk	2025-02-12	F	31	Penaforte
SEQ13_25_42	DENV2	2025-06-08	2025-06-11	F	25	Santana Do Cariri
SEQ13_25_44	DENV2	2025-06-03	2025-06-03	M	17	Santana Do Cariri
SEQ09_24_15	DENV1	2024-07-15	2024-07-17	F	83	Fortim
SEQ05_25_10	DENV2	2024-05-04	2024-05-06	F	14	Sao Benedito
SEQ05_25_18	DENV2	2024-05-20	2024-05-21	F	26	Barroquinha
SEQ05_25_19	DENV2	2024-05-18	2024-05-22	M	47	Jaguaribe
SEQ05_25_25	DENV2	2024-05-26	2024-05-27	F	18	Cruz

Sample ID	Serotype	Onset symptoms	Collection date	Sex	Age	Municipality
SEQ05_25_26	DENV2	2024-05-28	2024-05-29	F	64	Fortim
SEQ08_25_37	DENV2	Unk	2025-02-06	F	47	Penaforte
SEQ08_25_45	DENV2	Unk	2025-02-14	F	42	Penaforte
SEQ05_25_28	DENV2	2024-05-25	2024-05-28	F	50	Taua
SEQ05_25_34	DENV2	2025-01-13	2025-01-14	F	15	Penaforte
SEQ08_25_42	DENV2	Unk	2025-02-12	F	62	Barbalha
SEQ05_25_09	DENV2	2024-05-08	2024-05-08	M	9	Barroquinha
SEQ13_25_04	DENV1	2025-05-14	2025-05-16	M	13	Ico
SEQ09_25_08	DENV1	2025-04-25	2025-04-28	F	8	Redencao
SEQ13_25_27	DENV1	2025-06-13	2025-06-16	M	27	Fortim
SEQ09_24_20	DENV2	2024-07-27	2024-07-30	F	32	Fortim
SEQ09_24_02	DENV2	2024-06-10	2024-06-12	F	60	Cascavel
SEQ05_25_37	DENV1	2025-01-14	2025-01-17	M	18	Redencao
SEQ08_25_44	DENV2	Unk	2025-02-14	F	54	Penaforte
SEQ13_25_25	DENV1	2025-06-22	2025-06-24	M	24	Guaiuba
SEQ13_25_01	DENV1	2025-05-12	2025-05-14	F	10	Oros
SEQ13_25_32	DENV1	2025-06-22	2025-06-25	F	35	Fortaleza
SEQ09_24_21	DENV2	2024-07-31	2024-08-02	F	41	Graca
SEQ09_25_03	DENV1	Unk	2025-03-13	F	56	Jardim
SEQ09_25_13	DENV1	2025-05-06	2025-05-09	F	12	Redencao
SEQ09_25_16	DENV1	Unk	2025-03-21	M	24	Jardim
SEQ13_25_23	DENV1	2025-06-08	2025-06-11	M	18	Fortim
SEQ05_25_12	DENV1	2024-05-12	2024-05-13	M	27	Caninde
SEQ05_25_15	DENV1	2024-05-13	2024-05-16	F	44	Fortaleza
SEQ05_25_27	DENV1	2024-05-21	2024-05-22	F	37	Brejo Santo
SEQ09_25_06	DENV1	2025-04-20	2025-04-23	F	42	Acarape
SEQ13_25_31	DENV1	2025-05-29	2025-06-02	M	56	Oros
SEQ13_25_17	DENV1	2025-05-21	2025-05-23	F	77	Ico
SEQ09_25_12	DENV1	2025-05-02	2025-05-06	F	50	Jaguaribe
SEQ13_25_09	DENV1	2025-05-11	2025-05-15	F	22	Brejo Santo
SEQ09_25_05	DENV1	2025-05-03	2025-05-06	F	20	Redencao
SEQ09_25_10	DENV1	2025-04-25	2025-04-28	M	11	Acarape
SEQ05_25_17	DENV1	2024-05-14	2024-05-14	F	48	Cruz
SEQ05_25_33	DENV1	2025-01-09	2025-01-11	F	33	Penaforte
SEQ05_25_20	DENV1	2024-05-18	2024-05-24	M	20	Fortaleza
SEQ08_25_02	DENV1	2025-01-08	2025-01-09	M	67	Pedra Branca
SEQ05_24_32	DENV1	2024-05-23	2024-05-27	F	62	Fortaleza
SEQ05_25_23	DENV1	2024-05-24	2024-05-27	F	38	Potiretama
SEQ05_25_24	DENV1	2024-05-31	2024-06-02	F	17	Fortim
SEQ05_25_30	DENV1	2025-01-05	2025-01-08	F	15	Acarape
SEQ08_25_08	DENV1	2025-01-31	2025-02-04	M	14	Redencao
SEQ08_25_11	DENV1	2025-03-07	2025-03-09	F	14	Jaguaribe
SEQ08_25_23	DENV1	Unk	2025-03-10	M	16	Oros
SEQ13_25_02	DENV1	2025-05-12	2025-05-15	F	68	Jardim
SEQ13_25_05	DENV1	2025-05-17	2025-05-19	M	63	Oros
SEQ13_25_07	DENV1	2025-06-06	2025-06-10	F	48	Oros
SEQ13_25_10	DENV1	2025-04-21	2025-04-23	M	26	Barbalha
SEQ13_25_11	DENV1	2025-05-14	2025-05-16	F	18	Crato
SEQ13_25_12	DENV1	2025-05-16	2025-05-20	F	60	Barbalha
SEQ13_25_13	DENV1	2025-05-17	2025-05-19	F	16	Crato
SEQ13_25_16	DENV1	2025-05-29	2025-06-02	F	52	Oros
SEQ13_25_18	DENV1	2025-06-09	2025-06-11	M	10	Oros
SEQ13_25_20	DENV1	2025-06-15	2025-06-17	M	39	Fortim
SEQ13_25_29	DENV1	2025-05-12	2025-05-15	F	40	Barbalha
SEQ08_25_16	DENV1	2025-03-09	2025-03-13	F	37	Redencao
SEQ13_25_06	DENV1	2025-06-15	2025-06-17	F	28	Redencao
SEQ13_25_21	DENV1	2025-06-06	2025-06-09	M	8	Redencao
SEQ13_25_22	DENV1	2025-06-02	2025-06-04	F	18	Jardim
SEQ13_25_26	DENV1	2025-06-10	2025-06-11	F	14	Oros
SEQ05_24_13	DENV2	2024-05-01	2024-05-02	F	32	Graca
SEQ05_24_33	DENV1	2024-05-24	2024-05-28	F	38	Potiretama
SEQ05_24_27	DENV1	2024-05-14	2024-05-16	F	48	Cruz
SEQ05_24_16	DENV2	2024-04-30	2024-05-02	F	54	Graca
SEQ05_24_17	DENV2	2024-05-05	2024-05-09	F	11	Graca
SEQ09_24_04	Unk	2024-06-17	2024-06-17	M	24	Caucaia
SEQ09_24_26	Unk	Unk	2024-08-24	F	42	Milagres
SEQ05_25_03	DENV2	2024-05-01	2024-05-02	F	32	Graca
SEQ05_25_04	DENV2	2024-05-01	2024-05-02	M	33	Graca
SEQ05_25_05	DENV2	2024-04-30	2024-05-02	F	54	Graca

Sample ID	Serotype	Onset symptoms	Collection date	Sex	Age	Municipality
SEQ05_25_07	DENV2	2024-05-05	2024-05-08	F	11	Graca
SEQ05_25_08	DENV2	2024-05-06	2024-05-08	F	34	Graca
SEQ05_25_13	DENV1	2024-05-28	2024-05-28	F	37	Fortim
SEQ05_25_14	DENV1	2024-05-01	2024-05-09	M	28	Carnaubal
SEQ05_25_21	DENV2	2024-05-25	2024-05-07	F	27	Barroquinha
SEQ05_25_31	DENV1	2025-01-08	2025-01-09	M	67	Pedra Branca
SEQ13_25_19	DENV1	2025-06-03	2025-06-05	M	14	Cruz
SEQ09_25_01	DENV1	2025-03-23	2025-03-26	F	11	Acarape
SEQ09_25_02	DENV1	2025-03-31	2025-04-02	M	8	Acarape
SEQ13_25_30	DENV1	2025-06-07	2025-06-09	M	16	Redencao
SEQ13_25_24	DENV1	2025-06-13	2025-06-16	M	52	Senador Pompeu
SEQ05_25_02	DENV2	2024-04-28	2024-04-28	M	18	Barroquinha
SEQ05_25_32	DENV2	2025-01-08	2025-01-10	F	66	Penaforte

Legend: Unk, unknown. M, male. F, female.

Appendix Table 3. Genome assembly statistics for DENV-1-3 samples sequenced in this study (n=218)

Sample ID	Serotype	Ct	Coverage*	Average depth coverage*	Genotype	GenBank ID
SEQ03_24_13	DENV1	18.2	85.12	1647.61	DENV1 V_E1	TBC
SEQ03_24_07	DENV1	21.8	87.58	1806.42	DENV1 V_E1	TBC
SEQ03_24_01	DENV1	14	52.18	467.98	Unk	TBC
SEQ03_24_04	DENV1	16.8	36.91	191.35	Unk	TBC
SEQ03_24_03	DENV1	22.8	53.19	659.97	Unk	TBC
SEQ03_24_09	DENV1	21.5	90.97	3039.98	DENV1 V_E1	TBC
SEQ03_24_12	DENV1	21.5	91.91	2626.91	DENV1 V_E1	TBC
SEQ03_24_15	DENV1	18.7	18.59	45.99	Unk	TBC
SEQ03_24_05	DENV1	21.4	79.08	2590.04	DENV1 V_E1	TBC
SEQ03_24_06	DENV1	18.3	85.47	1970.05	DENV1 V_E1	TBC
SEQ03_24_08	DENV1	19.5	94.60	4759.35	Unk	TBC
SEQ03_24_10	DENV1	16.6	93.76	3568.81	DENV1 V_E1	TBC
SEQ03_24_11	DENV1	20.8	28.25	176.95	Unk	TBC
SEQ03_24_14	DENV1	22.5	92.58	3832.36	DENV1 V_E1	TBC
SEQ03_24_16	DENV1	22.3	95.69	4737.60	DENV1 V_E1	TBC
SEQ03_24_17	DENV1	24.5	93.44	4212.28	DENV1 V_E1	TBC
SEQ03_24_18	DENV1	16.7	92.44	2827.93	DENV1 V_E1	TBC
SEQ03_24_20	DENV1	18.1	89.01	2191.91	DENV1 V_E1	TBC
SEQ03_24_02	DENV1	20.4	81.14	1915.87	DENV1 V_E1	TBC
SEQ03_24_19	DENV1	18.3	79.30	1078.92	DENV1 V_E1	TBC
SEQ05_24_20	DENV2	Unk	14.39	57.41	Unk	TBC
SEQ13_25_14	DENV1	19.9	15.13	16.07	Unk	TBC
SEQ05_24_21	DENV2	Unk	20.55	42.29	Unk	TBC
SEQ05_24_14	DENV2	Unk	24.1	537.50	Unk	TBC
SEQ09_25_27	DENV2	19.9	24.97	3392.96	Unk	TBC
SEQ05_24_31	DENV1	Unk	25.82	397.88	Unk	TBC
SEQ05_24_39	DENV2	Unk	26.36	217.83	Unk	TBC
SEQ08_25_05	DENV1	18.8	34.17	1684.49	Unk	TBC
SEQ08_25_27	DENV2	27	34.63	224.26	Unk	TBC
SEQ09_25_22	DENV2	21.8	40.5	2334.90	Unk	TBC
SEQ08_25_07	DENV1	29.1	40.71	842.40	Unk	TBC
SEQ08_25_32	DENV2	31.9	41.27	1659.14	Unk	TBC
SEQ08_25_25	DENV2	32.4	42.3	1332.74	Unk	TBC
SEQ08_25_04	DENV1	21.1	50.21	1490.94	Unk	TBC
SEQ05_25_02	DENV2	22.2	52.59	2393.44	Unk	TBC
SEQ08_25_18	DENV1	32.9	54.59	569.98	Unk	TBC
SEQ05_24_24	DENV1	Unk	58.99	757.62	Unk	TBC
SEQ08_25_24	DENV2	31.3	59.89	1835.19	Unk	TBC
SEQ09_25_26	DENV2	18.6	60.1	2618.10	Unk	TBC
SEQ05_24_19	DENV2	Unk	61.81	842.63	Unk	TBC
SEQ13_25_46	DENV3	33	62.3	5970.90	DENV3 III_B3.2	TBC
SEQ05_25_39	DENV1	29.1	62.7	2402.37	Unk	TBC
SEQ09_24_10	DENV2	22.1	63.56	9877.38	Unk	TBC
SEQ08_25_06	DENV1	28.9	64.38	1060.90	Unk	TBC
SEQ13_25_43	DENV2	28.7	66.87	3111.24	Unk	TBC
SEQ09_24_14	DENV1	25	60.34	1017.87	Unk	TBC
SEQ09_25_25	DENV2	22.3	71.15	2881.58	DENV2 II_F1.1.2	TBC

Sample ID	Serotype	Ct	Coverage*	Average depth coverage*	Genotype	GenBank ID
SEQ05_25_11	DENV2	26.3	71.32	3678.75	DENV2 II_F1.1.2	TBC
SEQ09_24_08	DENV2	18.3	69.21	6319.92	Unk	TBC
SEQ05_24_26	DENV2	Unk	74.07	2553.23	DENV2 II_F1.1.2	TBC
SEQ13_25_40	DENV2	27.7	74.51	5281.32	DENV2 II_F1.1.2	TBC
SEQ09_25_21	DENV2	14.5	75.53	2538.53	DENV2 II_F1.1.2	TBC
SEQ09_24_28	DENV1	23.7	73.66	1646.42	DENV1 V_E1	TBC
SEQ09_25_19	DENV2	18	76.36	2495.85	DENV2 II_F1.1.2	TBC
SEQ09_24_05	DENV2	22	74.33	3.722.024	DENV2 II_F1.1.2	TBC
SEQ08_25_12	DENV1	32.7	78.22	1334.32	DENV1 V_E1	TBC
SEQ09_25_20	DENV2	23	79.05	2858.50	DENV2 II_F1.1.2	TBC
SEQ13_25_47	DENV3	32	79.17	4761.52	DENV3 III_B3.2	TBC
SEQ05_24_23	DENV1	Unk	80.76	5627.25	DENV1 V_E1	TBC
SEQ13_25_36	DENV2	22.3	80.76	2829.23	DENV2 II_F1.1.2	TBC
SEQ05_24_25	DENV1	Unk	80.99	3320.80	DENV1 V_E1	TBC
SEQ09_24_29	DENV1	28.2	72.35	3980.29	DENV1 V_E1	TBC
SEQ09_25_15	DENV1	19.2	81.89	1347.94	DENV1 V_E1	TBC
SEQ08_25_10	DENV1	29.2	82.49	643.43	DENV1 V_E1	TBC
SEQ08_25_35	DENV2	24.3	82.79	2502.25	DENV2 II_F1.1.2	TBC
SEQ08_25_30	DENV2	26	83.18	2331.88	DENV2 III_C1.1	TBC
SEQ13_25_34	DENV2	19.7	83.6	2888.16	DENV2 II_F1.1.2	TBC
SEQ09_25_23	DENV2	21.9	84.02	2472.39	DENV2 II_F1.1.2	TBC
SEQ09_24_25	DENV2	18.8	85.44	5049.34	DENV2 II_F1.1.5	TBC
SEQ09_24_01	DENV1	21.4	76.13	3430.64	DENV1 V_E1	TBC
SEQ09_24_22	DENV1	23.3	76.93	5489.15	DENV1 V_E1	TBC
SEQ13_25_24	DENV1	27.2	85.84	1721.58	DENV1 V_E1	TBC
SEQ09_25_24	DENV2	18.4	85.95	2933.73	DENV2 II_F1.1.2	TBC
SEQ13_25_30	DENV1	28.5	85.88	4180.13	DENV1 V_E1	TBC
SEQ05_24_35	DENV1	Unk	86.06	4886.39	DENV1 V_E1	TBC
SEQ09_24_17	DENV1	18.9	79.79	4545.54	DENV1 V_E1	TBC
SEQ08_25_33	DENV2	26	86.63	3203.35	DENV2 II_F1.1.2	TBC
SEQ05_24_37	DENV2	Unk	86.79	3592.93	DENV2 II_F1.1.2	TBC
SEQ08_25_28	DENV2	27	86.69	3028.39	DENV2 II_F1.1.2	TBC
SEQ05_24_36	DENV2	Unk	87.01	4102.63	DENV2 II_F1.1.2	TBC
SEQ05_24_15	DENV2	Unk	87.35	2713.69	DENV2 II_F1.1.2	TBC
SEQ05_24_28	DENV2	Unk	87.62	5680.46	DENV2 II_F1.1.2	TBC
SEQ05_24_29	DENV2	Unk	87.87	3740.97	DENV2 II_F1.1.2	TBC
SEQ13_25_41	DENV2	25.1	87.71	5153.86	DENV2 II_F1.1.2	TBC
SEQ05_24_18	DENV2	Unk	88.04	8423.57	DENV2 II_F1.1.2	TBC
SEQ09_25_14	DENV1	22	88.56	1614.58	DENV1 V_E1	TBC
SEQ05_25_06	DENV2	18.5	88.74	2717.30	DENV2 II_F1.1.2	TBC
SEQ05_24_30	DENV2	Unk	89.08	4577.78	DENV2 II_F1.1.2	TBC
SEQ05_25_35	DENV2	22.4	89.06	3735.71	DENV2 II_F1.1.2	TBC
SEQ05_24_34	DENV1	Unk	89.25	3721.86	DENV1 V_E1	TBC
SEQ13_25_45	DENV3	25.1	89.25	6934.99	DENV3 III_B3.2	TBC
SEQ05_25_01	DENV2	14.1	89.31	2153.83	DENV2 II_F1.1.2	TBC
SEQ09_24_11	DENV1	16.2	85.67	1144.37	DENV1 V_E1	TBC
SEQ09_24_27	DENV1	17	87.72	6361.91	DENV1 V_E1	TBC
SEQ09_24_30	DENV1	20.9	85.73	8180.59	DENV1 V_E1	TBC
SEQ09_24_16	DENV1	18	85.37	2765.16	DENV1 V_E1	TBC
SEQ09_24_03	DENV2	18.6	85.59	5217.34	DENV2 II_F1.1.2	TBC
SEQ05_25_38	DENV1	28.9	89.77	3486.48	DENV1 V_E1	TBC
SEQ08_25_40	DENV2	25	89.84	3606.81	DENV2 II_F1.1.2	TBC
SEQ09_24_19	DENV1	21.4	86.93	7403.23	DENV1 V_E1	TBC
SEQ09_24_23	DENV1	17.4	87.07	3660.34	DENV1 V_E1	TBC
SEQ05_25_29	DENV2	23.7	90.28	2709.88	DENV2 II_F1.1.2	TBC
SEQ08_25_38	DENV2	25	90.54	3748.97	DENV2 II_F1.1.2	TBC
SEQ08_25_19	DENV1	20.8	90.64	729.87	DENV1 V_E1	TBC
SEQ08_25_21	DENV1	23	90.64	789.01	DENV1 V_E1	TBC
SEQ05_25_16	DENV2	24.4	90.7	3621.36	DENV2 II_F1.1.2	TBC
SEQ08_25_01	DENV1	17.6	90.79	2478.84	DENV1 V_E1	TBC
SEQ08_25_09	DENV1	23.9	90.82	2962.41	DENV1 V_E1	TBC
SEQ08_25_22	DENV1	22	90.81	2569.21	DENV1 V_E1	TBC
SEQ08_25_14	DENV1	23.4	90.93	3193.77	DENV1 V_E1	TBC
SEQ09_25_09	DENV1	20	90.93	2986.06	DENV1 V_E1	TBC
SEQ13_25_03	DENV1	23.6	90.93	4485.03	DENV1 V_E1	TBC
SEQ09_24_09	DENV1	15.8	87.06	3714.01	DENV1 V_E1	TBC
SEQ13_25_28	DENV1	22.9	91.57	3171.82	DENV1 V_E1	TBC
SEQ09_25_17	DENV1	15	91.45	3758.84	DENV1 V_E1	TBC

Sample ID	Serotype	Ct	Coverage*	Average depth coverage*	Genotype	GenBank ID
SEQ09_24_24	DENV1	13.6	87.55	6852.78	DENV1 V_E1	TBC
SEQ09_25_18	DENV2	16	91.51	3573.4	DENV2 II_F1.1.2	TBC
SEQ09_24_07	DENV1	19.1	88.94	7478.37	DENV1 V_E1	TBC
SEQ09_24_18	DENV1	18.2	88.22	8199.27	DENV1 V_E1	TBC
SEQ05_25_32	DENV2	22.1	91.64	3520.36	DENV2 II_F1.1.2	TBC
SEQ13_25_37	DENV2	18.5	91.64	2717.58	DENV2 II_F1.1.2	TBC
SEQ08_25_36	DENV2	23	91.75	3768.55	DENV2 II_F1.1.2	TBC
SEQ09_25_07	DENV1	18.8	91.74	1990.60	DENV1 V_E1	TBC
SEQ13_25_08	DENV1	22.9	91.74	3775.84	DENV1 V_E1	TBC
SEQ08_25_26	DENV2	23	91.86	4944.44	DENV2 II_F1.1.2	TBC
SEQ09_25_04	DENV1	19	91.97	2352.06	DENV1 V_E1	TBC
SEQ09_25_11	DENV1	20.1	91.97	3105.44	DENV1 V_E1	TBC
SEQ05_25_22	DENV1	17.6	92.11	2753.04	DENV1 V_E1	TBC
SEQ09_24_06	DENV1	14.1	88.82	8147.38	DENV1 V_E1	TBC
SEQ09_24_12	DENV1	19	88.37	6111.31	DENV1 V_E1	TBC
SEQ13_25_35	DENV2	18.9	92.33	4271.61	DENV2 II_F1.1.2	TBC
SEQ05_24_38	DENV1	Unk	92.48	4257.88	DENV1 V_E1	TBC
SEQ08_25_29	DENV2	22	92.38	2875.69	DENV2 II_F1.1.2	TBC
SEQ08_25_41	DENV2	21	92.37	2361.07	DENV2 II_F1.1.2	TBC
SEQ13_25_38	DENV2	25	92.36	3373.22	DENV2 II_F1.1.2	TBC
SEQ13_25_39	DENV2	25.3	92.42	2048.35	DENV2 II_F1.1.2	TBC
SEQ08_25_34	DENV2	18	92.49	2868.35	DENV2 II_F1.1.2	TBC
SEQ08_25_43	DENV2	21	92.53	3387.94	DENV2 II_F1.1.2	TBC
SEQ09_24_13	DENV1	16.4	88.32	5674.95	DENV1 V_E1	TBC
SEQ13_25_33	DENV2	15.8	92.58	4318.83	Unk	TBC
SEQ08_25_03	DENV1	14.30	92.75	2634.92	DENV1 V_E1	TBC
SEQ08_25_39	DENV2	17	92.66	4072.63	DENV2 II_F1.1.2	TBC
SEQ13_25_42	DENV2	26.8	92.67	2812.72	DENV2 II_F1.1.2	TBC
SEQ13_25_44	DENV2	19.9	92.83	2564.18	DENV2 II_F1.1.2	TBC
SEQ09_24_15	DENV1	14.6	91.57	9417.18	DENV1 V_E1	TBC
SEQ05_25_10	DENV2	22.4	92.98	2997.68	DENV2 II_F1.1.2	TBC
SEQ05_25_18	DENV2	17.7	92.98	3032.72	DENV2 II_F1.1.2	TBC
SEQ05_25_19	DENV2	20.5	92.98	3058.85	DENV2 II_F1.1.2	TBC
SEQ05_25_25	DENV2	22.9	92.98	3600.50	DENV2 II_F1.1.2	TBC
SEQ05_25_26	DENV2	16.2	92.98	3441.81	DENV2 II_F1.1.2	TBC
SEQ08_25_37	DENV2	25	92.89	3766.41	DENV2 II_F1.1.2	TBC
SEQ08_25_45	DENV2	16	92.98	4116.94	DENV2 II_F1.1.2	TBC
SEQ05_25_28	DENV2	20	93	2680.06	DENV2 II_F1.1.2	TBC
SEQ05_25_34	DENV2	20.7	93.02	3116.54	DENV2 II_F1.1.2	TBC
SEQ08_25_42	DENV2	16	93.02	2955.83	DENV2 III_C1.1	TBC
SEQ05_25_09	DENV2	18.7	93.11	3327.38	DENV2 II_F1.1.2	TBC
SEQ13_25_04	DENV1	20.3	93.2	1970.32	DENV1 V_E1	TBC
SEQ09_25_08	DENV1	16.7	93.37	3208.06	DENV1 V_E1	TBC
SEQ13_25_27	DENV1	18.5	93.7	3884.22	DENV1 V_E1	TBC
SEQ09_24_20	DENV2	12.8	93.13	10849.99	DENV2 II_F1.1.5	TBC
SEQ09_24_02	DENV2	16.5	92.78	12251.31	DENV2 II_F1.1.2	TBC
SEQ05_25_37	DENV1	18.8	94.55	3556.95	DENV1 V_E1	TBC
SEQ08_25_44	DENV2	22	94.45	3360.27	DENV2 II_F1.1.2	TBC
SEQ13_25_25	DENV1	29.2	94.87	3548.69	DENV1 V_E1	TBC
SEQ13_25_01	DENV1	16.4	94.6	3267.53	DENV1 V_E1	TBC
SEQ13_25_32	DENV1	18.1	94.6	3220.78	DENV1 V_E1	TBC
SEQ09_24_21	DENV2	18.6	93.13	12123.97	DENV2 II_F1.1.2	TBC
SEQ09_25_03	DENV1	15	94.7	3343.17	DENV1 V_E1	TBC
SEQ09_25_13	DENV1	15.9	94.73	3240.67	DENV1 V_E1	TBC
SEQ09_25_16	DENV1	16	94.67	4060.86	DENV1 V_E1	TBC
SEQ13_25_23	DENV1	20.2	94.7	3166.80	DENV1 V_E1	TBC
SEQ05_25_12	DENV1	23.3	94.8	3554.03	DENV1 V_E1	TBC
SEQ05_25_15	DENV1	18.3	94.8	3724.07	DENV1 V_E1	TBC
SEQ05_25_27	DENV1	17.9	94.84	3709.18	DENV1 V_E1	TBC
SEQ09_25_06	DENV1	18.2	94.99	3109.51	DENV1 V_E1	TBC
SEQ13_25_31	DENV1	18.7	95	2714.55	Unk	TBC
SEQ13_25_17	DENV1	18.3	95.11	4110.60	DENV1 V_E1	TBC
SEQ09_25_12	DENV1	18.1	95.19	2925.79	DENV1 V_E1	TBC
SEQ13_25_09	DENV1	14.4	95.19	4447.60	DENV1 V_E1	TBC
SEQ09_25_05	DENV1	15.3	95.49	5265.78	DENV1 V_E1	TBC
SEQ09_25_10	DENV1	12.3	95.49	3449.46	DENV1 V_E1	TBC
SEQ05_25_17	DENV1	14.9	95.6	3850.16	DENV1 V_E1	TBC
SEQ05_25_33	DENV1	14.3	95.75	3484.17	DENV1 V_E1	TBC

Sample ID	Serotype	Ct	Coverage*	Average depth coverage*	Genotype	GenBank ID
SEQ05_25_20	DENV1	22	95.9	3330.89	DENV1 V_E1	TBC
SEQ08_25_02	DENV1	12.1	96.02	2990.29	DENV1 V_D1.1	TBC
SEQ05_24_32	DENV1	Unk	96.06	5066.08	DENV1 V_E1	TBC
SEQ05_25_23	DENV1	18.2	96.15	3607.21	DENV1 V_E1	TBC
SEQ05_25_24	DENV1	22.5	96.13	4197.19	DENV1 V_E1	TBC
SEQ05_25_30	DENV1	17.6	96.1	4053.81	DENV1 V_E1	TBC
SEQ08_25_08	DENV1	11	96.11	3313.91	DENV1 V_E1	TBC
SEQ08_25_11	DENV1	14.1	96.15	4067.77	DENV1 V_E1	TBC
SEQ08_25_23	DENV1	12	96.13	4375.01	DENV1 V_E1	TBC
SEQ13_25_02	DENV1	10.6	96.14	3516.06	DENV1 V_E1	TBC
SEQ13_25_05	DENV1	13.1	96.15	6325.63	DENV1 V_E1	TBC
SEQ13_25_07	DENV1	10.7	96.15	2922.88	DENV1 V_E1	TBC
SEQ13_25_10	DENV1	22.3	96.12	4363.70	DENV1 V_E1	TBC
SEQ13_25_11	DENV1	17.1	96.15	4223.01	DENV1 V_E1	TBC
SEQ13_25_12	DENV1	18.2	96.15	3804.65	DENV1 V_E1	TBC
SEQ13_25_13	DENV1	13.7	96.15	3432.90	DENV1 V_E1	TBC
SEQ13_25_16	DENV1	14.7	96.12	4139.31	DENV1 V_E1	TBC
SEQ13_25_18	DENV1	17.4	96.16	4236.21	DENV1 V_E1	TBC
SEQ13_25_20	DENV1	16.9	96.15	3643.15	DENV1 V_E1	TBC
SEQ13_25_29	DENV1	16.3	96.09	3190.04	DENV1 V_E1	TBC
SEQ08_25_16	DENV1	15.8	96.16	4002.28	DENV1 V_E1	TBC
SEQ13_25_06	DENV1	12.5	96.16	3896.35	DENV1 V_E1	TBC
SEQ13_25_21	DENV1	16.2	96.16	3544.30	DENV1 V_E1	TBC
SEQ13_25_22	DENV1	15	96.48	3583.08	DENV1 V_E1	TBC
SEQ13_25_26	DENV1	12.5	96.48	4475.24	DENV1 V_E1	TBC
SEQ05_24_13	DENV2	Unk	98.56	976.29	DENV2 II_F1.1.2	TBC
SEQ05_24_33	DENV1	Unk	98.91	3352.95	DENV1 V_E1	TBC
SEQ05_24_27	DENV1	Unk	99.24	8350.00	DENV1 V_E1	TBC
SEQ05_25_21	DENV2	17.3	93.02	3140.29	DENV2 II_F1.1.2	TBC
SEQ09_25_01	DENV1	21.5	90.93	874.57	DENV1 V_E1	TBC
SEQ09_25_02	DENV1	16.8	89.71	2706.96	DENV1 V_E1	TBC
SEQ05_25_02	DENV2	22.2	52.59	2393.44	Unk	TBC

Legend: Unk, unknown. Ct, RT-qPCR cycle quantification threshold value. *Genome and average depth coverage against to the DENV-1, DENV-2, and DENV-3 reference sequences (GenBank accessions NC_001477.1, NC_001474.2, NC_001475.2 for DENV-1, DENV-2, DENV-3 respectively).

Appendix Table 4. Tukey's Honest Significant Difference test of National Dengue incidence between age groups while accounting for the effect of sex between 2010-2025

Age groups	Age groups (years)							
(years)	0-9	10-19	20-29	30-39	40-49	50-59	60-69	≥70
0-9	-	0·05088^{ns}	0·15907^{ns}	0·19429^{ns}	0·16605^{ns}	0·13121^{ns}	0·44151^{ns}	0·68840^{ns}
10-19	-	-	0·96457^{ns}	0·92489^{ns}	0·95763^{ns}	0·98633^{ns}	0·59168^{ns}	0·36297^{ns}
20-29	-	-	-	0·.99999^{ns}	1·00000^{ns}	0·99999^{ns}	0·97424^{ns}	0·82594^{ns}
30-39	-	-	-	-	0·99999^{ns}	0·99994^{ns}	0·99155^{ns}	0·89339^{ns}
40-49	-	-	-	-	-	0·99999^{ns}	0·97913^{ns}	0·84161^{ns}
50-59	-	-	-	-	-	-	0·94274^{ns}	0·74958^{ns}
60-69	-	-	-	-	-	-	-	0·99922^{ns}
≥70	-	-	-	-	-	-	-	-

Statistical significance is ***p<0·001, **p<0·01, and *p<0·05; ns, not significant.

Appendix Table 5. Tukey's Honest Significant Difference test of National Dengue lab-confirmed cases between age groups while accounting for the effect of sex between 2010-2025

Age groups	Age groups (years)							
(years)	0-9	10-19	20-29	30-39	40-49	50-59	60-69	≥70
0-9	-	0·07428^{ns}	0·09807^{ns}	0·09640^{ns}	0·13174^{ns}	0·41281^{ns}	0·99999^{ns}	0·99614^{ns}
10-19	-	-	0·99999^{ns}	0·99999^{ns}	0·99934^{ns}	0·78943^{ns}	0·10092^{ns}	0·03531*
20-29	-	-	-	1·00000^{ns}	0·99999^{ns}	0·88828^{ns}	0·13344^{ns}	0·04614*
30-39	-	-	-	-	0·99999^{ns}	0·88302^{ns}	0·13117^{ns}	0·04538*
40-49	-	-	-	-	-	0·95813^{ns}	0·17912^{ns}	0·06151^{ns}
50-59	-	-	-	-	-	-	0·53311^{ns}	0·20072^{ns}
60-69	-	-	-	-	-	-	-	0·97475^{ns}
≥70	-	-	-	-	-	-	-	-

Statistical significance is ***p<0·001, **p<0·01, and *p<0·05; ns, not significant.

Appendix Table 6. Tukey's Honest Significant Difference test of state level dengue incidence in Ceará between age groups while accounting for the effect of sex between 2010-2025

Age groups	Age groups (years)							
(years)	0-9	10-19	20-29	30-39	40-49	50-59	60-69	≥70
0-9	-	0·12379^{ns}	0·46112^{ns}	0·71564^{ns}	0·90657^{ns}	0·99562^{ns}	0·99928^{ns}	0·86944^{ns}
10-19	-	-	0·91735^{ns}	0·69794^{ns}	0·47695^{ns}	0·26806^{ns}	0·06931^{ns}	0·02751*
20-29	-	-	-	0·99911^{ns}	0·96837^{ns}	0·79031^{ns}	0·27210^{ns}	0·10510^{ns}
30-39	-	-	-	-	0·99965^{ns}	0·96300^{ns}	0·46886^{ns}	0·19166^{ns}
40-49	-	-	-	-	-	0·99872^{ns}	0·68903^{ns}	0·31686^{ns}
50-59	-	-	-	-	-	-	0·92131^{ns}	0·54919^{ns}
60-69	-	-	-	-	-	-	-	0·98589^{ns}
≥70	-	-	-	-	-	-	-	-

Statistical significance is ***p<0·001, **p<0·01, and *p<0·05; ns, not significant.

Appendix Table 7. Tukey's Honest Significant Difference test of state level dengue lab-confirmed cases in Ceará between age groups while accounting for the effect of sex between 2010-2025

Age groups	Age groups (years)							
(years)	0-9	10-19	20-29	30-39	40-49	50-59	60-69	≥70
0-9	-	0·18019^{ns}	0·25792^{ns}	0·44940^{ns}	0·91471^{ns}	1·00000^{ns}	0·74170^{ns}	0·49364^{ns}
10-19	-	-	0·99997^{ns}	0·98492^{ns}	0·62188^{ns}	0·17913^{ns}	0·02856*	0·01681*
20-29	-	-	-	0·99906^{ns}	0·77833^{ns}	0·25644^{ns}	0·04037*	0·02341*
30-39	-	-	-	-	0·95902^{ns}	0·44714^{ns}	0·07298^{ns}	0·04140*
40-49	-	-	-	-	-	0·91315^{ns}	0·23667^{ns}	0·13308^{ns}
50-59	-	-	-	-	-	-	0·74420^{ns}	0·49603^{ns}
60-69	-	-	-	-	-	-	-	0·99929^{ns}
≥70	-	-	-	-	-	-	-	-

Statistical significance is ***p<0·001, **p<0·01, and *p<0·05; ns, not significant.

Appendix Table 8. Two-way ANOVA test of dengue frequency and incidence in Brazil and in Ceará between males and females

Incidence and cases	P value
National incidence	0.02473*
National cases	0.01765*
State-level incidence	0.00462**
State-level cases	0.00865**

Statistical significance is ***p<0·001, **p<0·01, and *p<0·05; ns, not significant.

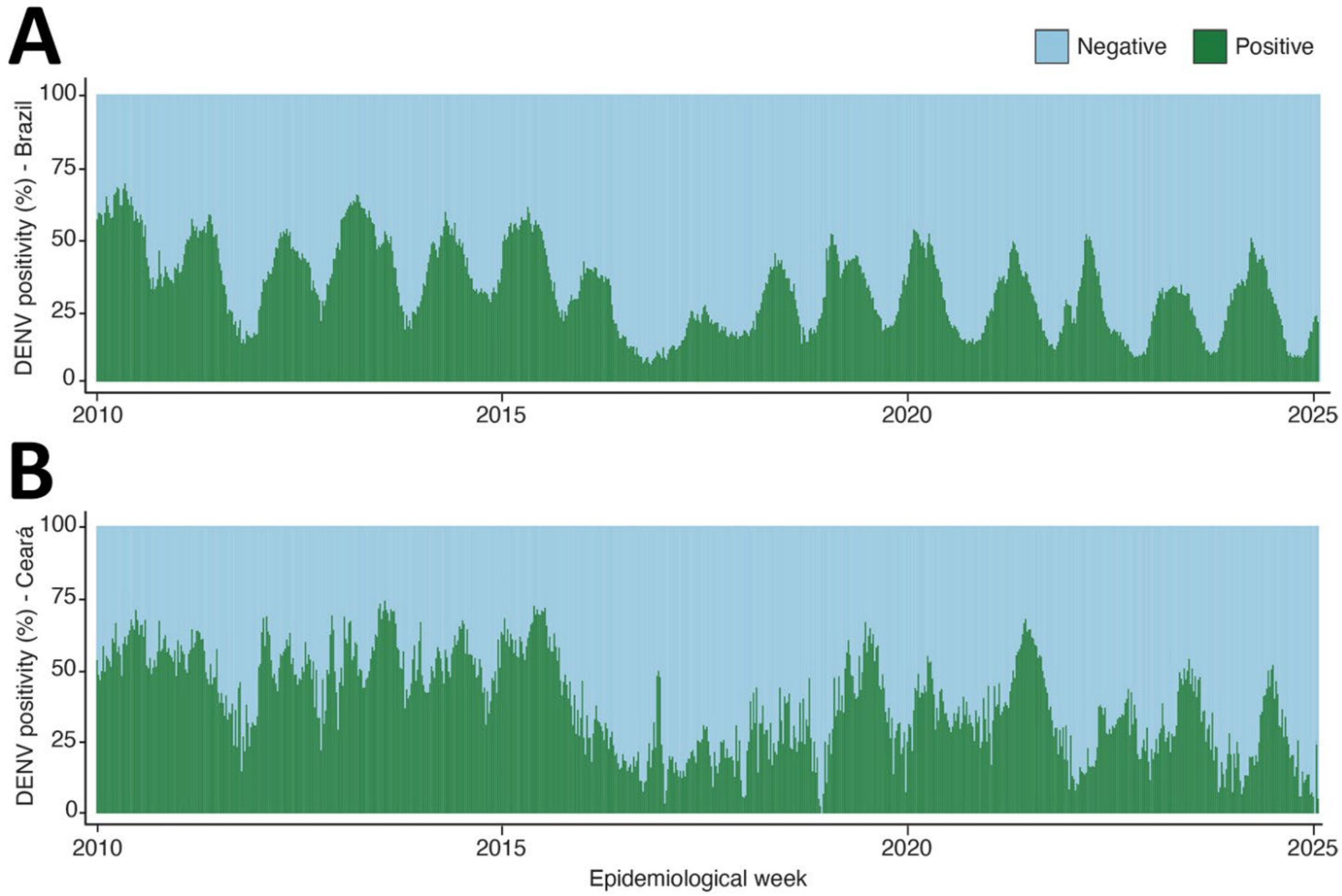

Appendix Figure 1. Weekly DENV test positivity (%) based on laboratory-confirmed dengue cases in Brazil (top) and Ceará state (bottom), epidemiological week 1 of 2010 (January 3-9) through epidemiological week 4 of 2025 (January 20-26).

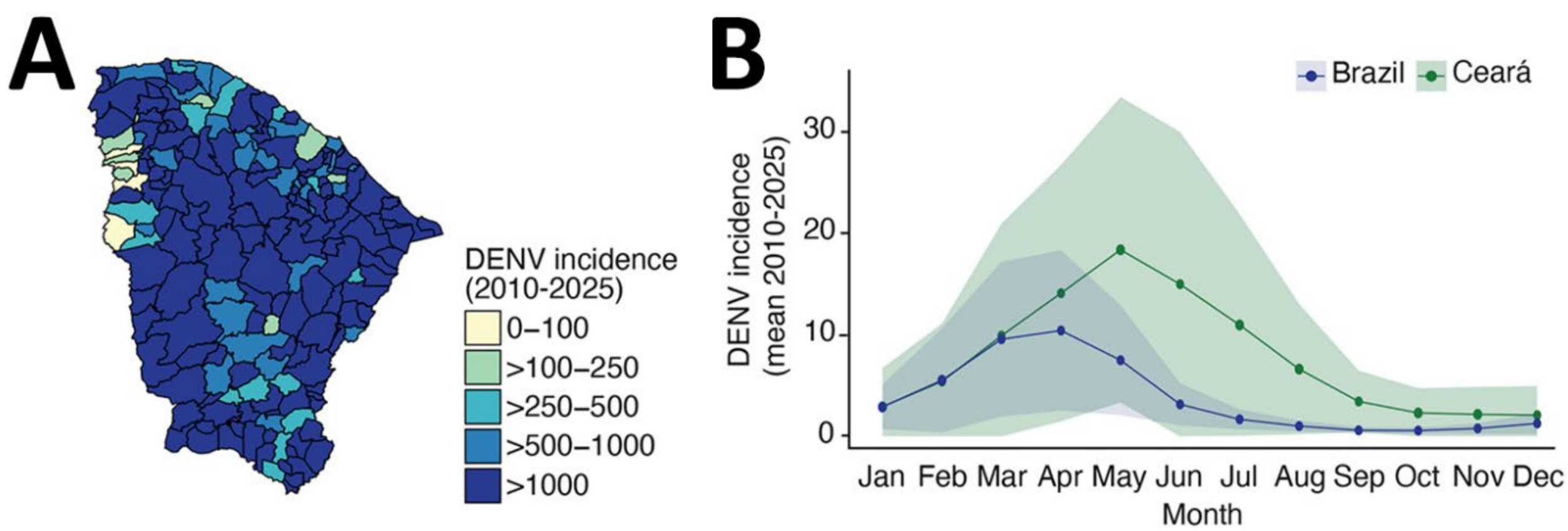

Appendix Figure 2. Spatial distribution and seasonal pattern of dengue incidence in Brazil and Ceará State, 2010-2025. A) Cumulative spatial distribution of dengue incidence per 100,000 inhabitants by municipality (n=184) in Ceará, 2010–2025. B) Monthly dengue incidence per 100,000 inhabitants in Brazil (right axis) and Ceará (left axis), 2010-2025; shaded areas represent ±1 standard deviation around the mean. DENV, dengue virus.

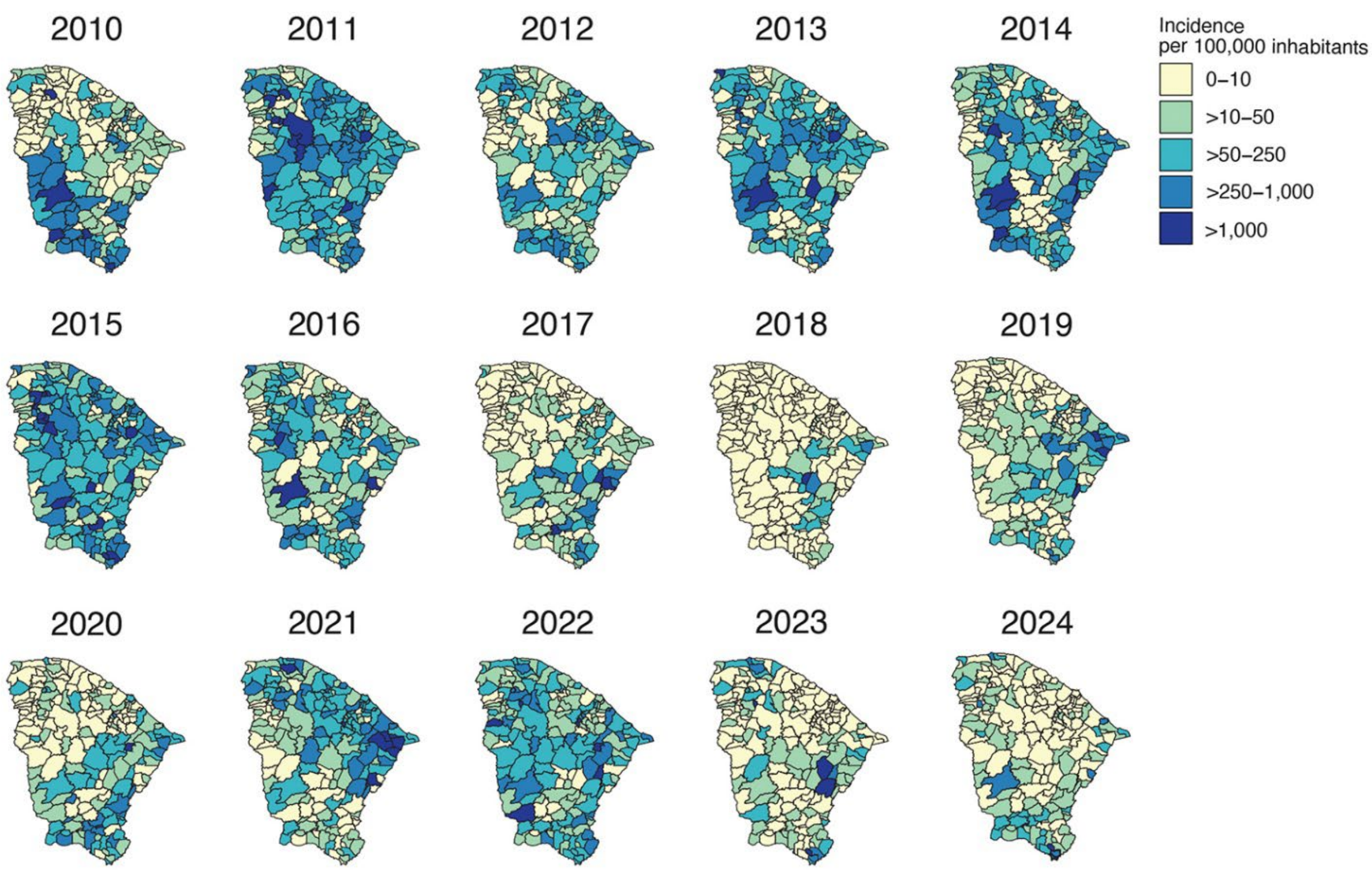

Appendix Figure 3. Annual spatial distribution of incidence of laboratory-confirmed dengue cases per 100,000 population by municipality (n = 184), Ceará state, Brazil, 2010–2025.

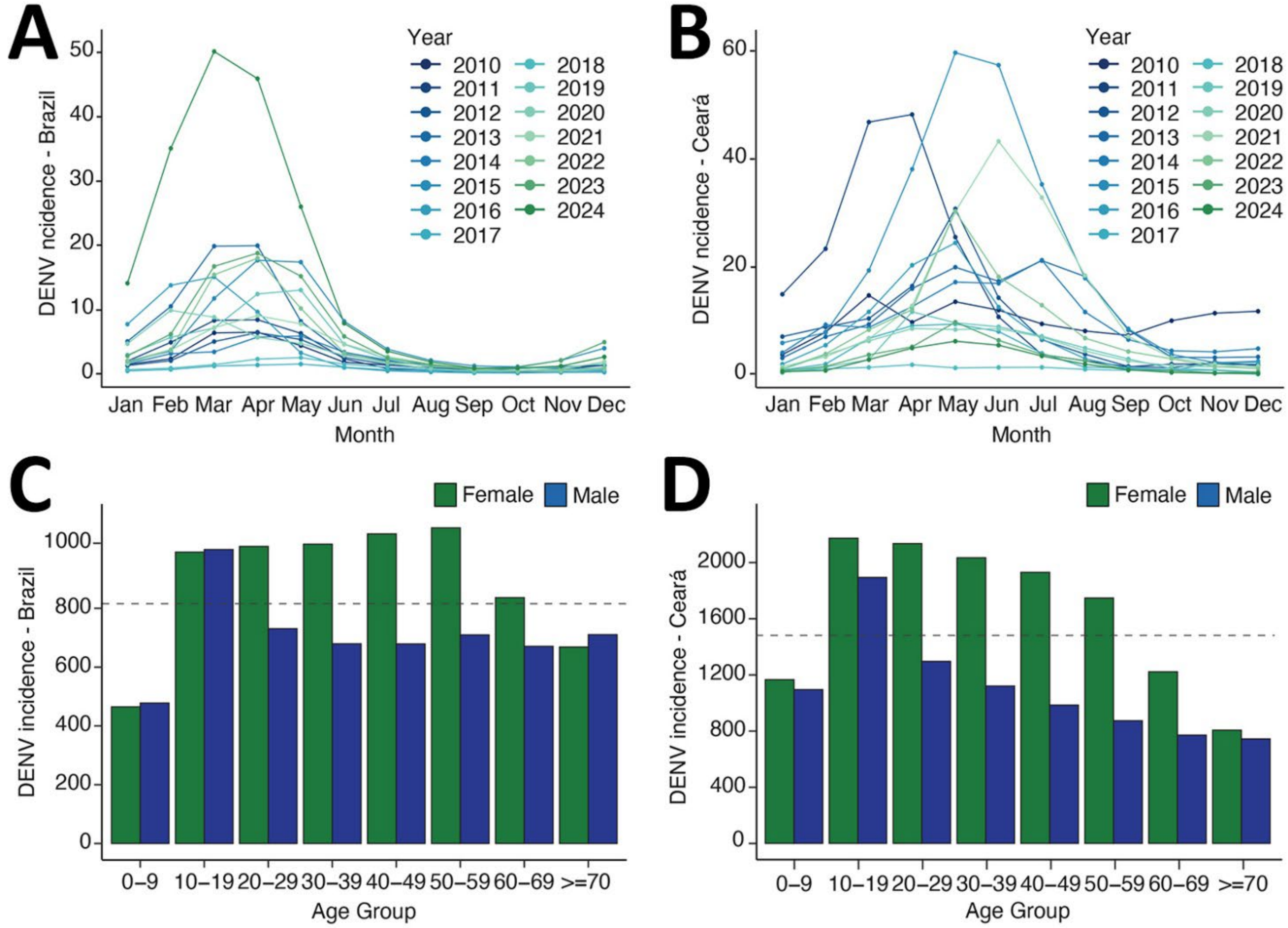

Appendix Figure 4. Seasonal and demographic patterns of dengue incidence in Brazil and Ceará state. A) Monthly incidence of laboratory-confirmed dengue cases in Brazil from 2010 to 2024. B) Monthly incidence of laboratory-confirmed dengue cases in Ceará State from 2010-2025. C) Age- and sex-stratified dengue incidence in Brazil (2010–2025); dashed line indicates the overall cumulative incidence of 815.9 cases per 100,000 inhabitants. D) Age- and sex-stratified dengue incidence in Ceará (2010-2025); dashed line indicates the overall cumulative incidence threshold of 1,500.9 cases per 100,000 inhabitants.

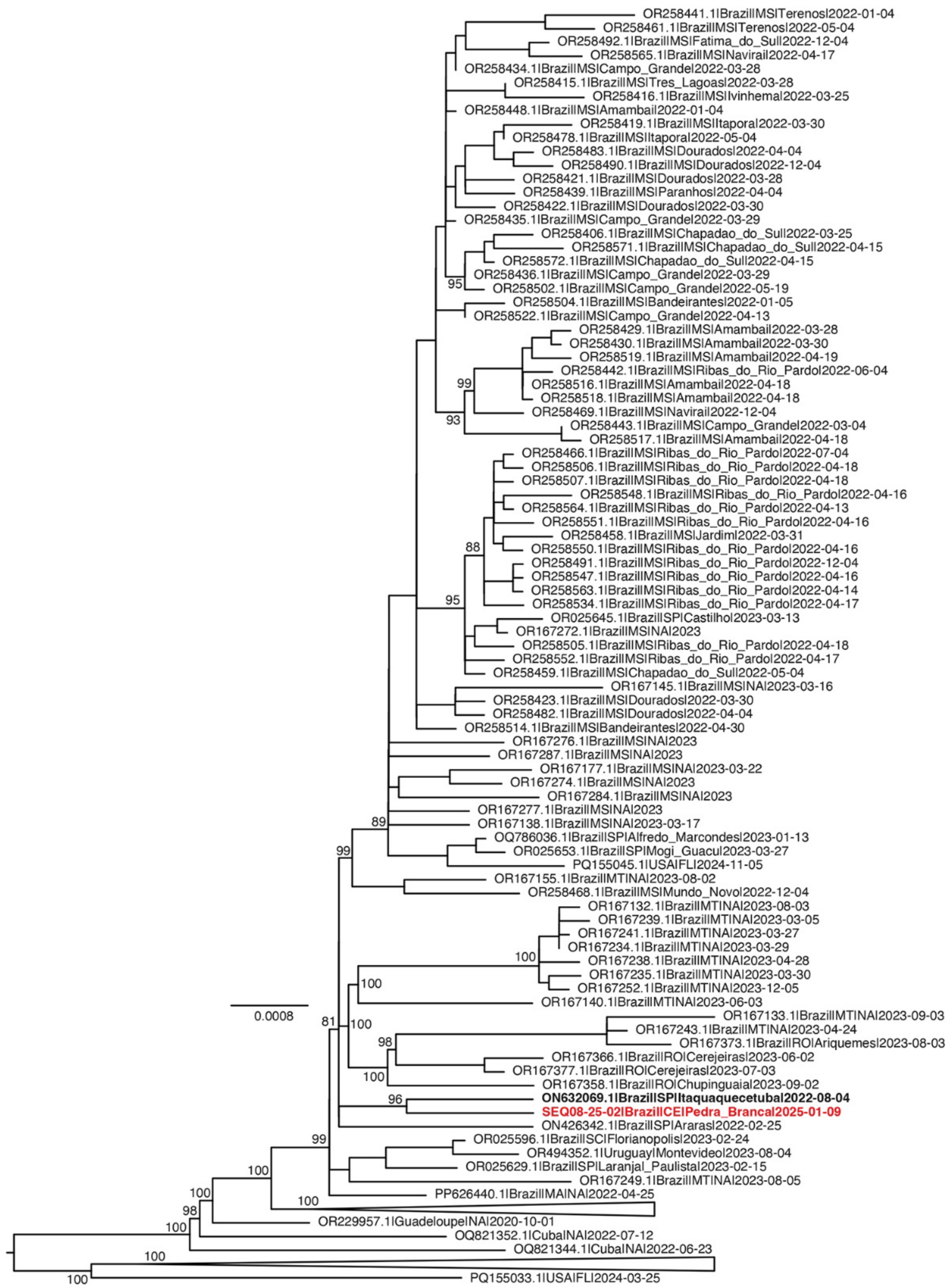

Appendix Figure 5. Maximum-likelihood phylogenetic analysis of DENV1 lineage V_D1.1 genomes (n=218), corresponding to the clade collapsed in Figure 2C. Genomes generated in this study (n=1) are highlighted in red. Tip labels indicate GenBank accession number, country, municipality (for Brazilian sequences), and year of collection. The tree is midpoint-rooted for clarity, and branch support was assessed with 1,000 ultrafast bootstrap replicates. The GTR+F+I+G4 model was applied. The scale bar represents substitutions per nucleotide site.

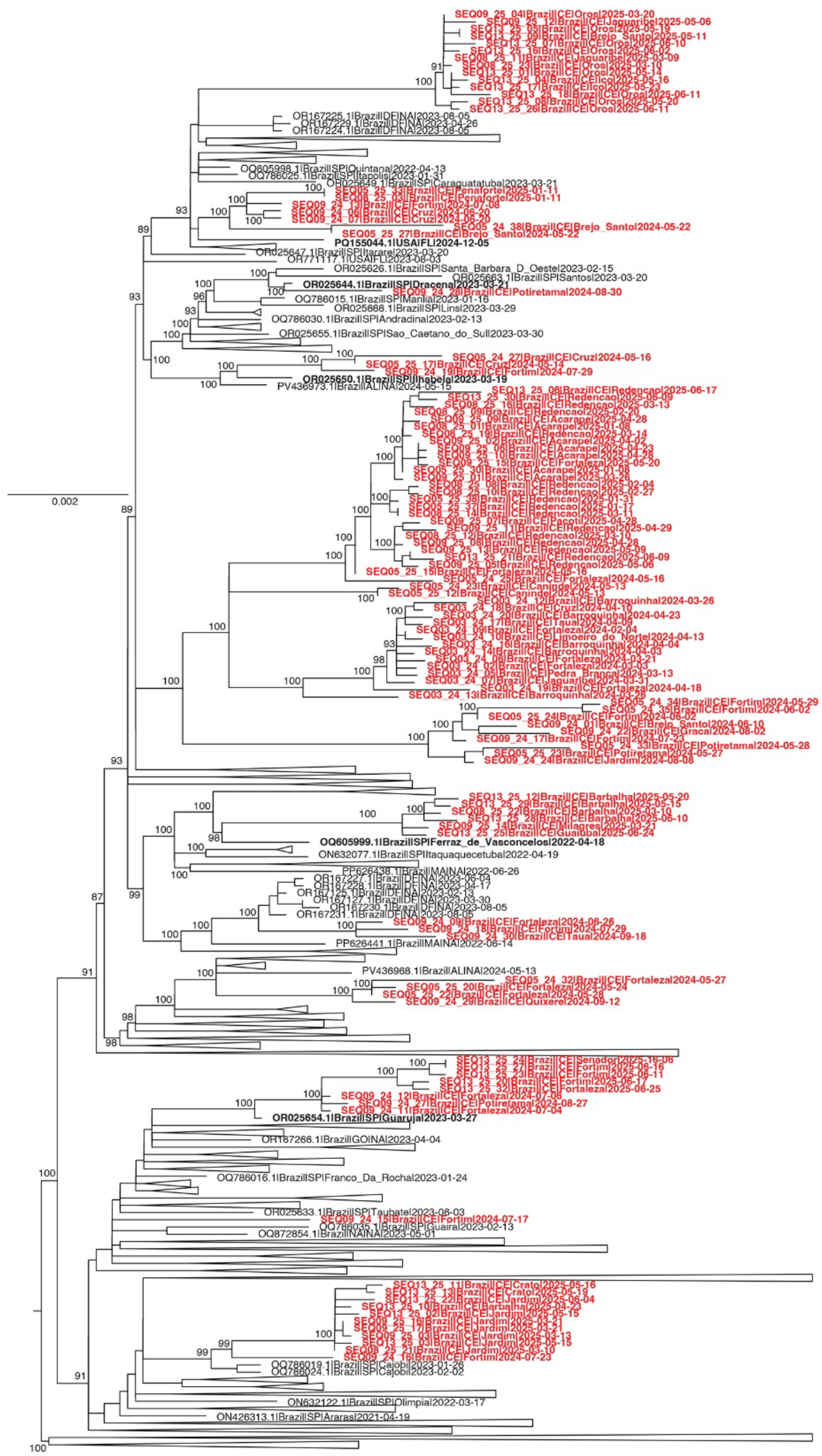

Appendix Figure 6. Maximum-likelihood phylogenetic analysis of DENV1 lineage V_E1 (n=791), corresponding to the clade collapsed in Figure 2C. Genomes generated in this study (n=111) are highlighted in red. Tip labels indicate GenBank accession number, country, municipality (for Brazilian sequences), and year of collection. The tree is midpoint-rooted for clarity, and branch support was assessed with 1,000 ultrafast bootstrap replicates. The GTR+F+I+G4 model was applied. The scale bar represents substitutions per nucleotide site.

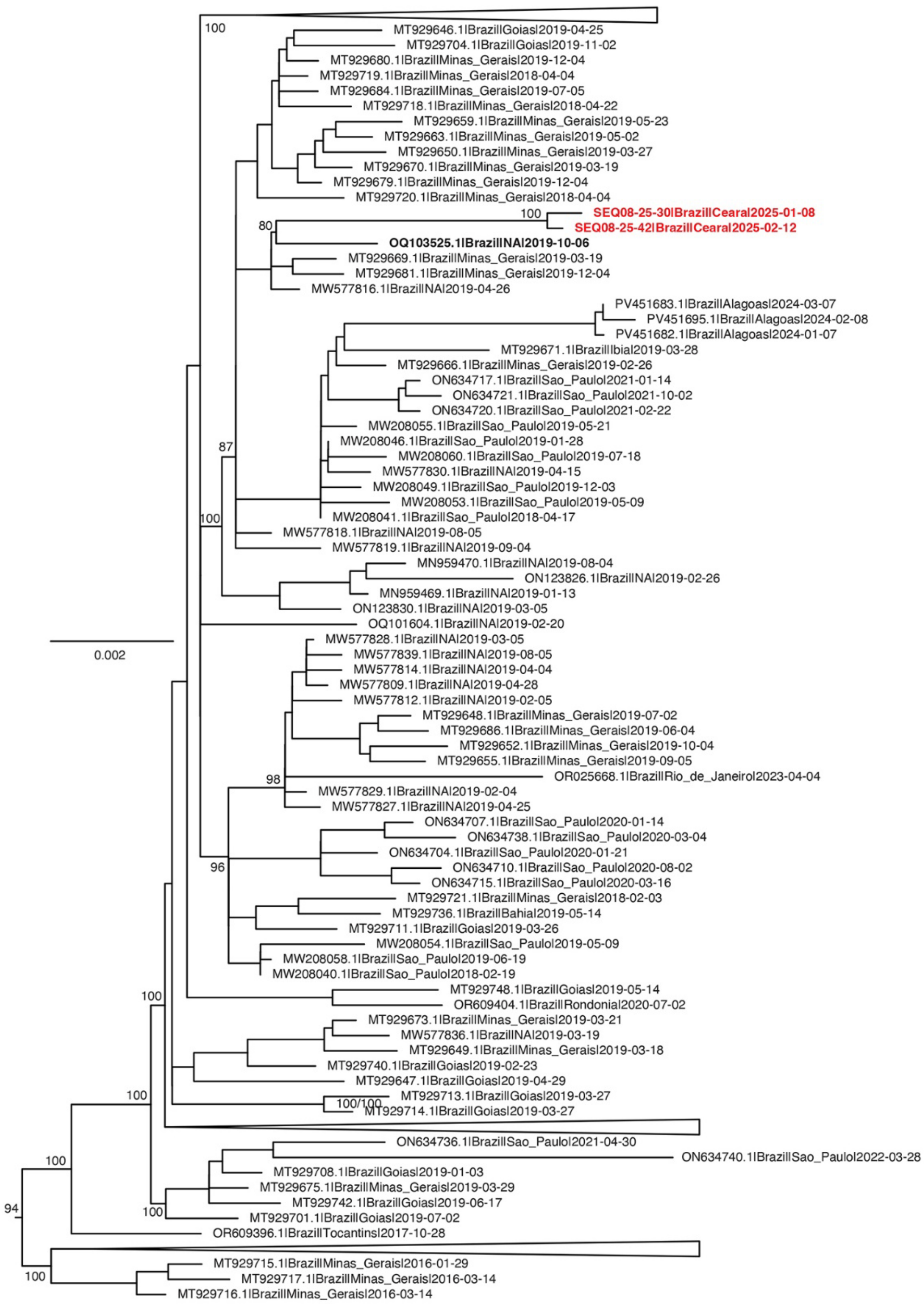

Appendix Figure 7. Maximum-likelihood phylogenetic analysis of DENV2 lineage III_C1.1 genomes (n=366), corresponding to the clade collapsed in Figure 2C. Genomes generated in this study (n=2) are highlighted in red. Tip labels indicate GenBank accession number, country, municipality (for Brazilian sequences), and year of collection. The tree is midpoint-rooted for clarity, and branch support was assessed with 1,000 ultrafast bootstrap replicates. The GTR+F+I+G4 model was applied. The scale bar represents substitutions per nucleotide site.

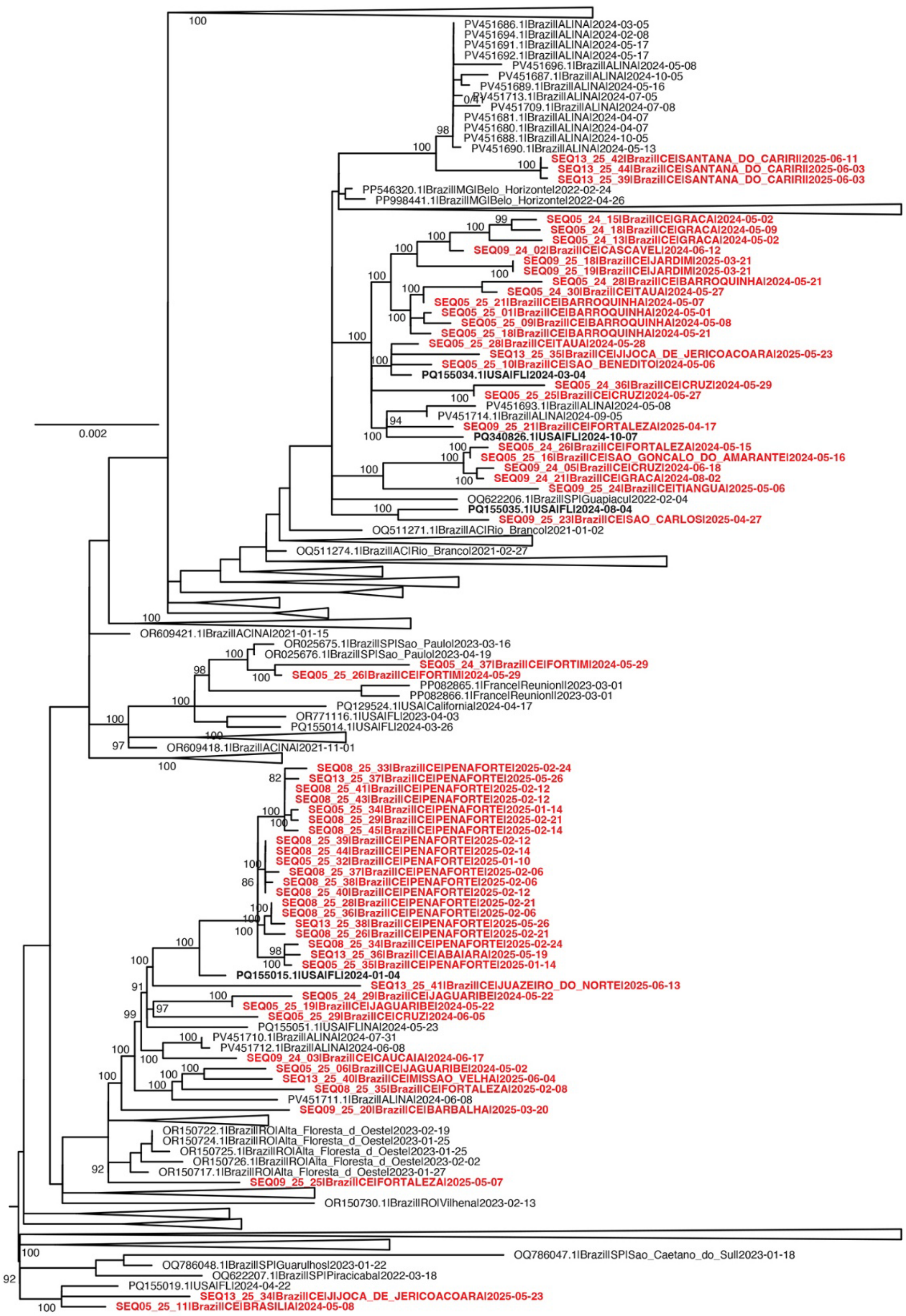

Appendix Figure 8. Maximum-likelihood phylogenetic analysis of DENV2 lineage II_F1.1.2 (n=499), corresponding to the clade collapsed in Figure 2C. Genomes generated in this study (n=61) are highlighted in red. Tip labels indicate GenBank accession number, country, municipality (for Brazilian sequences), and year of collection. The tree is midpoint-rooted for clarity, and branch support was assessed with 1,000 ultrafast bootstrap replicates. The GTR+F+I+G4 model was applied. The scale bar represents substitutions per nucleotide site.

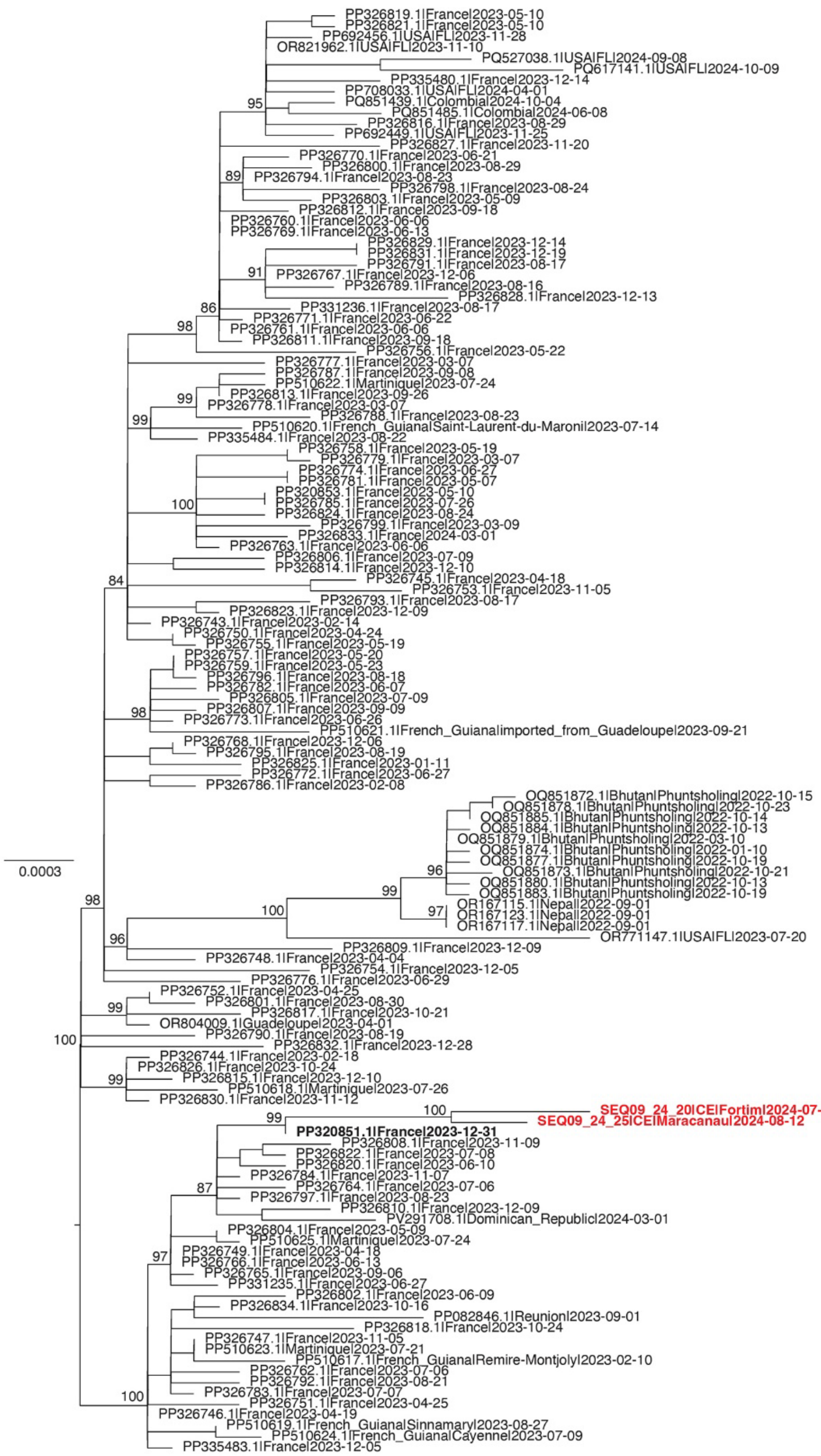

Appendix Figure 9. Maximum-likelihood phylogenetic analysis of DENV2 lineage II_F1.1.5 genomes (n=133), corresponding to the clade collapsed in Figure 2C. Genomes generated in this study (n=2) are highlighted in red. Tip labels indicate GenBank accession number, country, municipality (for Brazilian sequences), and year of collection. The tree is midpoint-rooted for clarity, and branch support was assessed with 1,000 ultrafast bootstrap replicates. The GTR+F+I+G4 model was applied. The scale bar represents substitutions per nucleotide site.

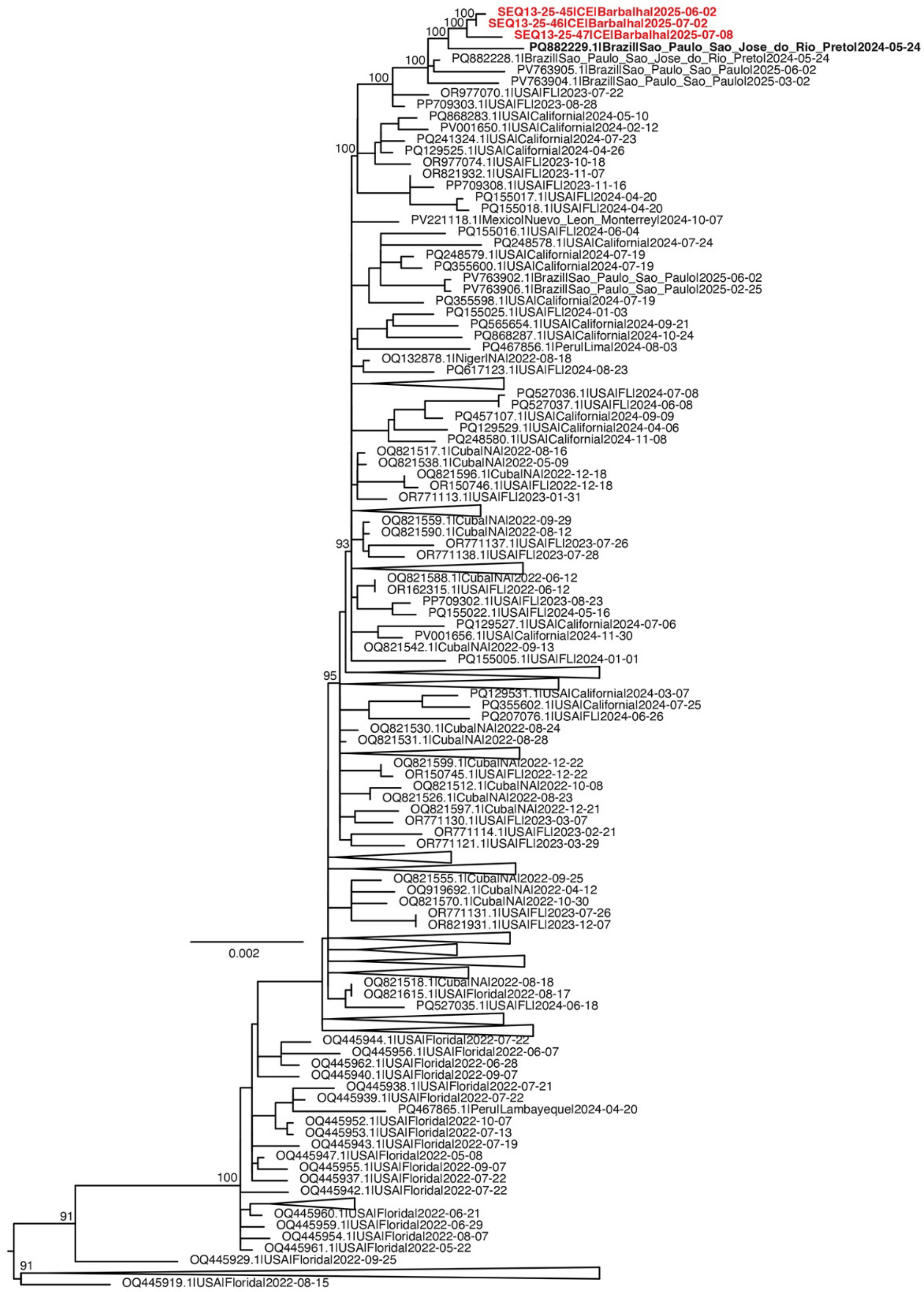

Appendix Figure 10. Maximum-likelihood phylogenetic analysis of DENV3 lineage III_B3.2 genomes (n=133), corresponding to the clade collapsed in Figure 2C. Genomes generated in this study (n=3) are highlighted in red. Tip labels indicate GenBank accession number, country, municipality (for Brazilian sequences), and year of collection. The tree is midpoint-rooted for clarity, and branch support was assessed with 1,000 ultrafast bootstrap replicates. The GTR+F+I+G4 model was applied. The scale bar represents substitutions per nucleotide site.
